# Orchestration of Autophagy and Senescence: Kinases Take the Center Stage

**DOI:** 10.3390/cells15131176

**Published:** 2026-06-28

**Authors:** Alakananda Basu

**Affiliations:** Department of Microbiology, Immunology and Genetics, University of North Texas Health Science Center, Fort Worth, TX 76107, USA; alakananda.basu@unthsc.edu

**Keywords:** PI3K/Akt/mTOR, AMPK, ULK, Ras/Raf/MEK/ERK, p38 MAPK, JNK, CDK, cancer, aging

## Abstract

**Highlights:**

**What are the main findings?**

- Kinases play a pivotal role in the complex interplay between autophagy and senescence.
- The mechanisms by which kinases affect autophagy and senescence involve mitochondrial and lysosomal dysfunctions, generation of reactive oxygen species, impairment of proteostasis and energy homeostasis, changes in spatial organization of autophagic compartments and alterations of the senescence-associated secretory phenotype which impacts the microenvironment.

**What is the implication of the main finding?**

- Kinases could be intervened to alter the detrimental effects of autophagy and senescence.
- A complete understanding of the mechanisms by which kinases regulate autophagy and senescence as well as proper use of methodologies and data interpretation are critical for exploiting kinases for therapeutic benefits.

**Abstract:**

Autophagy was originally identified as a survival mechanism to allow cells to survive under nutrient-deprived or stressful conditions whereas cellular senescence was considered a tumor-suppressive mechanism. Both processes can be induced by similar stimuli and can influence each other. There have been continued debates about whether they are causally linked, whether autophagy promotes or prevents senescence or if they are independent of each other. Protein kinases play integral roles in cell fate decision and have a major influence on both autophagy and senescence. While mechanistic target of rapamycin complex 1 is considered the master regulator of autophagy, it also influences senescence. Mitogen-activated protein kinases originally associated with senescence can regulate autophagy. While there have been numerous review articles on the interplay between autophagy and senescence, a comprehensive review on how various kinases participate in this interplay is lacking. The purpose of this review is to learn lessons from some old and recent studies to understand how kinases contribute to this changing field. Since both autophagy and senescence can have beneficial and detrimental effects and kinases are important drug targets, insights regarding how kinases orchestrate these two processes should help develop therapeutic strategies to treat diseases, such as aging and cancer.

## 1. Introduction

Cells communicate with external factors and internal signaling molecules to regulate cellular functions. Under basal state, processes, such as autophagy and cellular senescence, maintain homeostasis, but when cells are challenged with stresses, they are activated [1]. Autophagy or self-cannibalism is an evolutionarily conserved catabolic process which involves degradation and recycling of cellular components to survive under starvation or stressful conditions [2]. The elimination of long-lived, misfolded, non-functional proteins and damaged organelles by autophagy serves an important function in quality control. On the other hand, cellular senescence prevents accumulation of damaged cells by inducing stable cell cycle arrest [3].

Autophagy can cause both selective and non-selective degradation of cellular constituents via three different routes [4]. Macroautophagy, often used interchangeably with autophagy, could be non-selective bulk degradation of cellular components or selective degradation of specific organelles, such as mitochondria, endoplasmic reticulum, Golgi and nucleus [5]. During macroautophagy, the cytosolic materials are enclosed in double membrane vesicles called autophagosomes which fuse with lysosomes to form autolysomes for degradation of the cargo and its constituents. During microautophagy, cargos are directly recognized by lysosomal invagination [5]. Chaperone-mediated autophagy (CMA) (see list of abbreviations) involves highly selective degradation of individual non-functional proteins containing the KFERQ (LysPheGluArgGln)-targeting motif in the lysosomes [5,6].

Cellular senescence is also categorized based on the types of stress. Telomerase attrition during normal process of aging can cause replicative senescence (RS) [7] whereas dysfunction of the immune system with aging is categorized as immonosenescence [8]. Cellular senescence induced prematurely in response to various stresses, such as DNA damage, chemotherapeutic drugs, reactive oxygen species and oncogene activation, is collectively known as stress-induced premature senescence or SIPS [9]. It is also categorized based on specific types of stress. For example, activation of oncogenes, such as Ras, BRAF and Myc, can trigger oncogene-induced senescence (OIS) to serve as a barrier to tumorigenesis [10]. Many therapeutic agents, such as chemotherapy, radiotherapy and immunotherapy, can induce premature senescence known as therapy-induced senescence (TIS) [3]. Since the mechanisms of senescence may differ with different oncogenes, they are further specified as Ras-induced senescence (RIS) [11], Akt-induced senescence (AIS) [12] or PTEN-loss-induced cellular senescence (PICS) [13]. Senescence can exert both beneficial and detrimental effects [14,15,16,17].

Both autophagy and senescence can cause tumor suppression and tumor promotion [3,18,19,20,21,22]. Both processes can also be triggered by similar stress inducers, such as oncogenes, tumor suppressor proteins, DNA damage and oxidative stress, suggesting they are closely linked [23]. Thus, there have been intense investigations of the interconnection between autophagy and senescence [6,24,25,26,27,28,29,30,31].

Protein kinases play fundamental roles in signal transduction and cell regulation and are important targets for cancer therapy. Several protein kinases have been associated with the initiation and execution of autophagy as well as senescence. Although there are several review articles on how a particular kinase regulates either autophagy or senescence, or their involvement in a particular disease type [23,30,32,33,34,35,36], a comprehensive review on the mechanisms by which different kinases regulate both processes is lacking. The purpose of this review article is to explore the literature on dual regulation of autophagy and senescence by kinases regardless of the model system. Although the focus of this review is not on clinical studies, a mechanistic understanding of how kinases orchestrate both processes is essential prior to targeting them for therapeutic benefits. Thus, we believe this review would benefit not only researchers but also clinicians engaged in age-related disorders.

## 2. Kinases Involved in Autophagy and Senescence

This section introduces several kinases that have been implicated in both autophagy and senescence.

**Figure 1:**
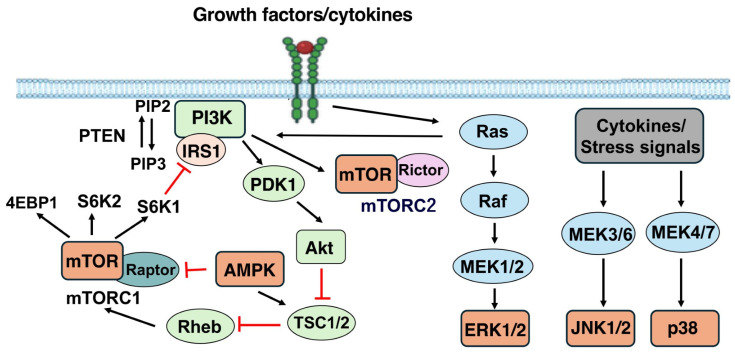
A simplified diagram of mTOR, AMPK and MAPK kinase signaling. See text for details. The growth factor receptor icon is from Biorender.com.

**PI3K/Akt/mTOR**: Phosphatidylinositol 3-kinases (PI3K), a family of lipid kinases, are categorized into three groups. Class I PI3K plays an important role in growth factor signaling whereas class III PI3K functions in membrane trafficking [37]. Class I PI3K is activated in response to hormones, growth factors and cytokines and phosphorylates phosphatidylinositol 4,5-bisphosphate (PIP2) to phosphatidylinositol 3,4,5-trisphosphate (PIP3) (Figure 1) [37]. Phosphorylation of PDK1 (3-phosphoinositide-dependent kinase 1) by PI3K activates Akt by phosphorylating at the Thr308 site via PDK1 whereas dephosphorylation of PIP3 by the tumor suppressor PTEN (phosphatase and tensin homolog) inactivates PI3K [38].

Akt acts both upstream and downstream of mTOR (mechanistic target of rapamycin) which exists as two complexes—mTORC1 and mTORC2 [39]. The primary distinction between the complexes is the binding of raptor to mTORC1 and rictor to mTORC2. Akt activates mTORC1 by phosphorylating TSC2 (tuberous sclerosis complex 2) at Ser939 and Thr1462, a negative regulator of mTORC1 [40]. On the other hand, mTORC2 activates Akt by phosphorylating Akt at the Ser473 site. mTORC1 mediates its function via its downstream targets S6K1/2 (ribosomal S6 kinase 1 and 2) and 4E-BP1 (eukaryotic translation initiation factor 4E binding protein 1) [41]. However, S6K1 can inhibit PI3K/Akt signaling via a negative feedback loop involving IRS1. Thus, persistent inhibition of mTORC1 may in fact activate PI3K/Akt via S6K1. The PI3K/Akt/mTOR signaling pathway is frequently deregulated in many diseases, including cancer [37].

**AMPK:** AMP-activated protein kinase (AMPK) senses the energy needs of the cell and is activated during ATP depletion, nutrient deprivation and oxidative stress [42]. It plays important roles in regulating mitochondrial function and cellular metabolism. It activates catabolism and inhibits anabolic processes [43]. AMPK is activated by phosphorylation at Thr172 by upstream kinases, such as LKB1 (liver kinase B1) and CaMKK (calcium/calmodulin-dependent protein kinase kinase) [42]. It can inhibit mTORC1 by phosphorylating TSC2 at Thr1227 and SerS1345 and raptor at Ser722 and Ser792 (Figure 1) [44,45]. AMPK can also phosphorylate the tumor suppressor protein p53 at Ser15 to induce cell cycle arrest [46].

**MAPK:** Mitogen-activated protein kinases (MAPK) play major roles in transmitting extracellular signals to a variety of intracellular events [47]. The classical MAPKs can be categorized into three major groups: extracellular signal-regulated kinases 1/2 (ERK1/2), c-Jun amino (N)-terminal kinases (JNK) and p38 MAPK. ERK1 and -2 are activated by growth factors, mitogens and cytokines via a cascade of kinases. Activation of Ras by receptor tyrosine kinases activates Raf which in turn activates MEK1/2 culminating in the activation of ERK1/2 (Figure 1) [47]. JNK and p38 MAPK are primarily activated by cellular stress such as reactive oxygen species (ROS), DNA damaging agents, radiation, cytokines, etc. JNK, also known as stress-activated protein kinase or SAPK, consists of three members, JNK1, JNK2 and JNK3, whereas p38 MAPK consists of four isoforms, α, β, γ and δ [47].

**CDK**: The activation of cycle-dependent kinases (CDK), a family of serine/threonine protein kinases, is critical for cell cycle progression [48]. Different cyclins bind to their catalytic partners to trigger various phases of the cell cycle. While activation of cyclin D-CDK4/6 is necessary to enter the cell cycle, cyclin E-CDK2 and cyclin A-CDK2 are required for the progression of cells through the G1/S, S and G2/M phase. Conversely, inhibition of CDKs by cyclin-dependent kinase inhibitors halts cell cycle progression [48].

## 3. Regulation of Autophagy by Kinases

The process of macroautophagy (referred to as autophagy) has been studied extensively [49,50]. It involves several steps, including the induction of the initiation complex, nucleation of phagophore formation, elongation of the phagophore to form autophagosome, maturation of autophagosome, fusion of autophagosome with lysosome to form autophagolysosome or autolysosome, degradation of the cargos by lysosomal hydrolases and recycling of the degraded materials. Each of the steps starting from initiation to completion of autophagic flux is regulated by kinases. This section includes a brief description of how kinases mentioned above regulate various steps of autophagy.

**Induction:** During the first step, an initiation complex consisting of ULK (Unc51-like kinase)-1 (Atg1 in yeast), FIP200 (FAK family kinase-interacting protein of 200 kDa), autophagy-related proteins, Atg101 and Atg13 is formed. ULK1 is a serine/threonine kinase which is phosphorylated by upstream kinases and can undergo autophosphorylation. ULK1 can also phosphorylate several proteins to regulate various steps of the autophagy process. There are several members in the ULK family, including ULK1, ULK2, ULK3 and ULK4 [51]. While ULK2 is believed to be functionally redundant with ULK1, it also exhibits distinct functions [52].

The induction of autophagy is regulated primarily by three different kinases—mTORC1, AMPK and ULK1 [53,54,55]. mTORC1 is considered the master regulator of autophagy during starvation-induced autophagy. During nutrient-rich conditions, it inhibits autophagy by phosphorylating ULK1 at Ser758 and Ser638 and Atg13 at Ser258 [54]. While it was originally believed that AMPK can activate autophagy directly by phosphorylating ULK1 at Ser556 or indirectly by inhibiting mTORC1 by phosphorylating raptor or TSC2, a recent study challenged that notion [56]. During energy crisis, such as mitochondrial dysfunction or glucose deprivation, activation of AMPK causes phosphorylation of ULK1 at Ser556 and Thr660, stabilizing its association with ULK1. This allows ULK1 to retain mTORC1-mediated phosphorylation at the Ser758 site inhibiting autophagy induction during starvation. Stabilization of AMPK-mediated initiation complex prevents abrupt induction of autophagy during energy crisis and protects against caspase-mediated degradation of autophagy machinery until homeostasis is restored and autophagy can resume [56]. ULK1 is also activated by autophosphorylation at Thr180 [57].

CDK1 activity is required to trigger mitosis. mTORC1 is inhibited during mitosis due to CDK-dependent phosphorylation of raptor [58]. However, autophagy initiation is repressed even though mTORC1 is inhibited during mitosis since CDK1 substitutes for mTORC1 and phosphorylates ULK1, ATG13 and ATG14 at the mTORC1 sites inhibiting autophagy during mitosis independent of mTORC1.

**Nucleation:** In the second step, the lipid kinase activity of class III PI3K (PI3KC3) is necessary to generate PI3P at the phagophore. It forms two complexes [54]. Once ULK1 is activated, the initiation complex recruits PI3KC3 complex 1 (PI3KC3-C1) which is composed of the catalytic subunit VPS34 (vacuolar protein sorting 34), regulatory subunit VPS14, beclin 1, ATG14 and AMBRA1 (activating molecule in beclin 1-regulated autophagy protein 1) to initiate nucleation.

This step is regulated by several kinases. mTORC1 phosphorylates Atg14 to inhibit PI3KC3-C1 activity whereas ULK1/2 phosphorylates beclin 1 and Atg14 to activate the lipid kinase VPS34 [59,60]. mTORC1 directly phosphorylates AMBRA1 at Ser 52 to negatively regulate autophagy [61]. CDK1 phosphorylates VPS34 at Thr159 to inhibit its interaction with beclin 1 [62] and phosphorylates Atg14 to repress autophagy during mitosis [58]. JNK1 induces autophagy by phosphorylating Bcl-2, causing its dissociation from beclin 1 [63]. MEK/ERK can induce protective autophagy by acting downstream of AMPK and upstream of mTORC1 [64].

**Autophagosome formation and maturation:** Atg9A, which is present at the plasma membrane and trans-Golgi network, serves as a source of the membrane for delivering lipids from different organelles to the initiation complex [65] and for membrane expansion [66]. Phospholipid transfer is facilitated by Atg2 [2]. Phosphorylation of Atg9A by ULK1 is necessary for Atg9A trafficking—translocation of Atg9 vesicles to the initiation complex [67,68]. It is also phosphorylated by AMPK [69] and p38 MAPK [70] to initiate autophagosome formation.

Autophagosome formation and maturation require PI3KC3 complex II (PI3KC3-CII) in which Atg14 of PI3KC3-C1 is replaced with UVRAG (UV radiation resistance-associated gene protein). UVRAG interacts with beclin 1 to promote autophagosome formation and with VPS34 to facilitate autophagosome maturation which requires conjugation of LC3 to the autophagosomal membrane [71]. Kinetics of autophagosome formation of osteosarcoma cells revealed that activation of AMPK during glucose starvation reduces autophagosome maturation by inhibiting tethering of donor membranes to the phagophore [72]. However, how AMPK-mediated phosphorylation affects phagophore tethering remains to be established.

The expansion of the phagophore requires two ubiquitin conjugation systems: Atg8 (LC3)-ATG7-ATG3 and ATG12-ATG5-ATG16L. Both Akt and JNK increased the expression of ATG8 or LC3 [73,74]. Processing of inactive Pro-LC3 to LC3 requires ATG4B prior to lipidation of LC3-I to LC3-II and its insertion in the autophagosomal membrane. While ULK1 inhibited ATG4B activity, Akt2 was shown to activate processing of LC3 by ATG4B [75]. Phosphorylation of Atg5 by p38 MAPK caused inhibition of starvation-induced autophagy [76]. Activation of JNK1/2 caused upregulation of Atg7 [77] whereas JNK2 caused upregulation of beclin 1 and Atg5 [63]. Phosphorylation of ATG16L by ULK1 induced its cleavage [78].

**Formation of autolysosomes, degradation and recycling:** Autophagosomes fuse with lysosomes to degrade the cargo and enclosed materials. Phosphorylation of UVRAG by mTORC1 inhibits the fusion of autophagosomes with lysosomes [79,80]. Replenishment of lysosomes is needed to sustain autophagy. TFEB (transcription factor EB) is required for the transcription of genes involved in autophagy and lysosomal biogenesis [81]. Phosphorylation of TFEB by mTORC1 retains it in the cytoplasm and prevents nuclear translocation to transcribe genes necessary for lysosome biogenesis [54].

Kinases also regulate selective autophagy. Phosphorylation of ULK1 by PKCα enhances chaperone-mediated autophagy [82] whereas phosphorylation of Atg13 by ULK1 causes clearance of damaged mitochondria by inducing mitophagy [83].

## 4. Regulation of Cellular Senescence by Kinases

Cellular senescence (hereinafter referred to as senescence) is characterized by stable cell cycle arrest. Senescent cells are metabolically active and exhibit enlarged flatten morphology, chromatin modifications, altered metabolism and acquisition of the senescence-associated secretory phenotype (SASP) [3,84]. The following section includes a discussion of the mechanisms by which different kinases contribute to senescence from selected articles so that it is easier to comprehend the interplay between autophagy and senescence by kinases. Additional information can be found in several review articles [32,34,85,86,87,88,89,90,91,92,93].

### 4.1. Cycle-Dependent Kinases (CDK)

Acquisition of a stable cell cycle arrest is the hallmark of cellular senescence. Inactivation of the tumor suppressor protein Rb is necessary for cell cycle progression [94]. Rb is phosphorylated by CDK4/6 and subsequently by CDK2 and CDK1 to release the transcription factor E2F which transcribes genes necessary for DNA replication during the S phase of the cell cycle. Inhibition of CDK4/6 by p16^INK4A^ activates Rb and restores its ability to inhibit cell cycle progression [94]. Thus, the p16^INK4A^/Rb pathway has been associated with cellular senescence [95].

Activation of the tumor suppressor protein p53 is necessary to halt the cell cycle when cells are subjected to stress signals, such as DNA damage [94,96]. In normal cells, half-life of p53 is very short due to its degradation via the ubiquitin ligase MDM2 (murine double minute 2) [96]. In response to DNA damage, activation of ATM, ATR and Chk1/Chk2 phosphorylate and stabilize p53 causing an increase in its transcriptional activity [97]. Depending on the intensity and duration of the stress signals, p53 can trigger transient cell cycle arrest, senescence or apoptosis [98].

p53 is a transcription factor which transcribes CDK inhibitor p21 to inhibit cell cycle progression [99]. Thus, the p53/p21 pathway is also important to trigger cellular senescence. In response to oncogenic signals, p53 is stabilized by p19^ARF^ encoded by the *INK4A* gene but has no relationship with p16^INK4A^. p19^ARF^ stabilizes p53 by inhibiting MDM2 [100]. Because of the central role of CDKs in senescence, there are numerous articles that discuss their involvement in senescence [86,88,89,90,92].

### 4.2. Ras/Raf/MEK/ERK Pathway

Table 1 summarizes how MAPK signaling regulates senescence in response to different senescent inducers and under different experimental conditions.

While telomere attrition is associated with replicative senescence, Serrano et al. demonstrated that oncogenic Ras can also induce senescence in primary human (IMR90) or rodent (REF52) fibroblasts [120]. Oncogenic H-RasV12 caused a transient increase in cell proliferation in low serum followed by the induction of senescence in IMR90 and mouse embryo fibroblasts (MEF) [101]. Upregulation of both p53/p21 and p16 via MEK which acts downstream of Raf (Figure 1), but not PI3K/Akt, was responsible for the induction of senescence and knockout of either *p53* or *p16^INK4A^* was sufficient to promote tumorigenesis in mice. However, constitutively active (CA) oncogenic Raf induced senescence in non-immortalized IMR90 lung fibroblasts via MEK/ERK-mediated upregulation of p16 independent of p53/p21 [102]. Since MEK/ERK acts downstream of Ras and Raf (Figure 1), the lack of involvement of p53/p21 in this study [102] is not clear. One possibility is that while Lin et al. [101] used p53 or p21 null MEFs, Zhu et al. [102] used the E6 oncoprotein to inactivate p53. Thus, the methodological differences could influence the outcome.

CA-MEK1 also induced senescence in non-immortalized intestinal epithelial cells (IEC) via activation of both the p53/p21 and p16/Rb pathway but promoted growth in immortalized IECs [103], suggesting an additional mechanism operates in immortalized cells to override premature senescence. ERK1b, an alternatively spliced form of ERK1, was attributed to the growth-promoting activity of CA-MEK in immortalized IECs since ERK1b was highly induced and phosphorylated only in CA-MEK expressing immortalized but not non-immortalized IECs and phosphorylation of ERK1b correlated with cell proliferation and transformation of immortalized cells.

An unbiased siRNA screen combined with proteomics further established the importance of the ERK pathway during oncogenic Ras-induced senescence. The mechanism by which ERK (especially ERK2) induced senescence in oncogenic Ras expressing IMR90 fibroblasts, human mammary epithelial cells (HMEC) and mouse embryo fibroblasts (MEFs) involved proteosomal degradation of selected proteins, such as c-MYC, STAT3 and HSP27 [104]. Senescence-associated protein degradation (SAPD) induced mitochondrial dysfunction, generation of ROS and DNA damage response, causing induction of senescence via p53/p21 and p16/Rb pathways. Knockdown of ERK2 was sufficient to reverse senescence and promote transformation in Ras oncogene-expressing cells whereas depletion of SAPD targets could restore senescence. An important finding from this study is that the intensity of ERK signaling is a deciding factor of whether ERK would promote tumor suppression or promote transformation and its clinical relevance was tested in several prostate cancers. ERK is known to induce cell proliferation in response to growth factors (Figure 1). However, both ERK activity and senescence marker p16 were high and SAPD target STAT3 was low in benign prostatic hyperplasia (BPH). In contrast, ERK activity was extremely low, and the STAT3 level was high in aggressive prostate tumors. These results support the notion that ERK-mediated selective protein degradation suppresses tumor development by inducing senescence. In fact, ERK activity is extremely low in pancreatic cancers that harbor mutant Ras. Thus, depleting SAPD targets rather than ERK could provide a better therapeutic approach.

Subsequent studies confirmed the involvement of ERK2 but not ERK1 in Ras oncogene-induced senescence [105]. ERK2 cooperated with both p38 MAPK and p70 ribosomal S6 kinase-1 (S6K1), a downstream target of mTORC1. Silencing of ERK2 caused a decrease in the p38/AP1/cJun/ETS pathway which regulates p16 transcription and the TSC2/mTORC1/p706K1 pathway which regulates p19^ARF^ translation, an upstream regulator of p53. However, the direct involvement of p38 or mTORC1 on ERK2-mediated upregulation of p16^INK4A^ and p19^ARF^ was not investigated.

To address variations in ERK activity in benign versus malignant tumors and heterogeneity in oncogene-induced senescence, Chen et al. [121] performed live cell imaging in non-transformed hTERT-immortalized retinal pigment epithelial (RPE) cells overexpressing oncogenic Raf (BRAF^V600E^). The choice between cell proliferation and senescence was decided by the activation status of ERK. An intermediate ERK activity promoted cell proliferation, higher activity induced senescence and lower activity contributed to Raf oncogene-induced transformation due to temporal induction of distinct classes of genes dictated by the ERK activity. The biphasic effect of MAPK was also observed during conversion of MEFs to motor neurons. Lende-Dorn and colleagues used a chemogenetic approach to alter MAPK activity in cells expressing intermediate and high levels of H-RAS^G12V^. Since MAPK acts downstream of RAS (Figure 1), MAPK activity depends on the strength of RAS signaling. High levels of oncogenic RAS enhanced MAPK activity driving cells to senescence [122].

While the intensity of ERK activity decided whether ERK would promote cell proliferation or induce senescence, the duration of ERK signaling determined the maintenance of senescence [106]. A senescence restriction point (SeRP) acts as a checkpoint to integrate duration and intensity of oncogenic stress. ERK signaling was only required to be sustained until the senescence restriction point, after which ERK activity was no longer needed [106]. ERK2 engaged a network of transcription factors, including ETV4, RUNX1, OCT1 and MAFB, which made chromatin more accessible, facilitating the expression of proinflammatory genes. Chromatin opening served as a commitment to oncogene-induced senescence downstream of ERK signaling. The clinical relevance of the transcription factor network was validated in pancreatic cancer. ETV4 could restore senescence in a pancreatic cell line that escaped therapy-induced senescence. Moreover, the levels of ETV4 and RUNX1 were high in benign pancreatic lesions but downregulated in pancreatic adenocarcinoma. Since ERK has dual functions on tumor suppression and tumor promotion depending on its activation status, it may be better to increase chromatin accessibility which acts downstream of ERK as a potential therapeutic strategy to suppress tumors.

### 4.3. JNK

Unlike ERK, ablation/inhibition of JNK1/2 induced premature senescence. Phosphorylation of c-Jun by JNK activates the AP1 transcription factor which negatively regulates p53. Therefore, knockout of JNK caused upregulation of p53 and induction of p53/21-dependent senescence [107]. Inhibition/depletion of JNK but not ERK or p38 MAPK also induced senescence in primary human embryonic fibroblasts (HEF) as wells as in MCF-7 breast cancer and H460 lung carcinoma cells. JNK also sensitized MCF-7 cells to ionizing radiation-induced senescence [108]. The mechanism by which JNK inhibited senescence involved phosphorylation of Bcl-2 which maintains mitochondrial integrity. Inhibition of Bcl-2 phosphorylation by the JNK inhibitor increased mitochondrial ROS which triggered DNA damage response causing induction of p53, increase in p21 and dephosphorylation of Rb resulting in the induction of senescence.

In contrast to above studies, the activation of JNK was associated with the induction of senescence in a mouse model of prostate cancer in which PTEN was deleted [123]. Inactivation/loss of PTEN causes development of prostatic intraepithelial neoplasia (PIN) but not invasive cancer due to induction of senescence [124]. Deletion of JNK in PTEN-deleted mice contributed to the development of invasive adenocarcinoma by suppressing senescence [123].

Senescence-associated stable cell cycle arrest is followed by the release of SASP factors. These two steps may be regulated via distinct mechanisms. When senescence was induced by ionizing radiation, anticancer drug etoposide or oxidative stress in IMR90 cells, JNK was activated by ROS generated from dysfunctional mitochondria in senescent cells causing accumulation of cytoplasmic chromatin fragments (CCFs) which triggered SASP via the cGAS-STING pathway [109]. Inhibition/depletion of JNK in senescent fibroblasts decreased the accumulation of CCFs and the release of SASP cytokines but failed to suppress ROS production or rescue cell cycle arrest, suggesting that JNK acting downstream of mitochondrial ROS induced SASP but not cell cycle arrest [109].

JNK was also shown to play distinct roles during the induction and maintenance of senescence during topoisomerase II inhibitors-induced DNA damage in osteosarcoma U2OS cells [110]. Following treatment with low doses of doxorubicin or mitoxantrone, JNK/ERK initiated senescence through AP1/cJun causing cell cycle arrest at G2/M and an increase in p21 and SA-β-gal activity. This induction of senescence during the early phase could be reversed by the JNK and ERK inhibitors or the inhibition/KD of cJun, but the p38 inhibitor had no effect. At later time points, JNK/ERK signaling committed to senescence by releasing SASP factors, such as IL-6 and IL-8 [110]. This temporal effect of JNK was observed only in response to topoisomerase II inhibitors but not in response to cisplatin or neocarzinostatin (NCS). This study is in contrast to an earlier study where inhibition of JNK induced p53-mediated senescence via AP1 in MEFs [107]. The differences between these studies are in the use of cell type (cancer versus normal cells) and senescence inducer (DNA damage by chemotherapeutic drugs). Thus, any therapeutic strategies targeting the JNK or ERK pathway should consider cellular context as well as their temporal roles in regulating senescence.

### 4.4. p38 MAPK

p38 was activated by chronic stress and played a causal role during oncogene-induced, oxidative stress-induced and replicative senescence [111]. However, p38 was not directly activated by the initial senescence stimuli but by the activation of the ERK pathway and remained activated even after the withdrawal of the senescence inducers. Chronic activation of p38 caused Rb-dependent p53-independent cell cycle arrest but the increase in SA-β-gal activity by p38 in senescent cells was independent of both Rb and p53 [111]. Activation of p38 played an essential role in maintaining stable cell cycle arrest during DNA damage-induced and telomere-dependent and -independent senescence acting downstream of *CDKN1A* which encodes for p21 [112]. Prolonged activation of p21 following DNA damage caused mitochondrial dysfunction resulting in an increased production of ROS which sustained DNA damage response (DDR) via a positive feedback loop involving p53-p21-GADD45-p38-TGFβ, thus maintaining persistent growth arrest and senescent phenotype in vitro and in vivo [112].

Activation of p38 could contribute to SASP independent of p53/p21 upregulation in normal human fibroblasts [113]. In fact, p53 inhibited p38 MAPK activity and SASP induction. Delayed and sustained activation of p38 during DNA damage- and oncogene-induced senescence was required for the secretion of some SASP components, including chemokines, cytokines and growth factors and contributed to the invasion of MDA-MB-231 cells in a paracrine manner. A high level of constitutively active (CA)-p38α was necessary and sufficient for SASP induction independent of DDR but required NF-κB activity which acted synergistically with p38. However, in response to oncogenic Ras or irradiation, p38 required DDR to induce SASP [113]. p38 also induced SASP during replicative senescence via its downstream target MK2 (MAPK-activated protein kinase 2) [125]. Two p38 inhibitors UR-13756 and BIRB 796 with better selectivity and specificity compared to SB230058 as well as MK2 inhibitors attenuated secretion of proinflammatory SASP cytokine IL-6. Using a two-step protocol of squamous cell carcinoma, Campisi and colleagues [114] demonstrated that the induction of senescence by activation of p38 and ERK1/2 could contribute to tumor promotion but not tumor initiation. Treatment with the anticancer drug doxorubicin induced senescence and SASP which in turn activated p38 and ERK1/2 in skin tumors enhancing tumor growth and progression of benign papilloma to carcinoma whereas elimination of senescent cells inhibited p38 activity and prevented cancer progression. These results suggest that senescence induced by chemotherapeutic agents could contribute to tumor development by activating p38 MAPK and stimulating SASP and provides an explanation for cancer recurrence and secondary tumor development following treatment with chemotherapeutic drugs.

p38 activation also contributed to senescence when fatty acid synthesis was inhibited by the knockdown (KD) of either acetyl-CoA carboxylase 1 (ACC1), the rate-limiting enzyme of fatty acid biosynthesis, or fatty acid synthase [115]. ACC1 level decreased during replicative and oxidative stress-induced senescence and KD of ACC1 increased ROS production resulting in the activation of p38. While inhibition of p38-MAPK suppressed ACC1 KD-induced increase in SA-β-gal staining, it failed to restore cell proliferation, suggesting that p38 mediates only part of the senescence program during inhibition of lipid synthesis [115]. Another mechanism by which p38α contributed to cellular senescence involved transcriptional repression of *hTERT* via Sp1/HDAC1 (histone deacetylase 1) [116]. p38 activity increased during serial passaging of normal human fibroblasts. Activation of p38 by the cytokine IL-1β was associated with repression of *hTERT* transcription whereas inhibition of p38 by DN-p38α or a pharmacological inhibitor counteracted repression of *hTERT* expression in A549 lung cancer cells. Decrease in *hTERT* expression was sufficient to induce senescence, suggesting that p38 acted upstream of *hTERT* [116].

p38 could induce senescence independent of DDR via accumulation of lamin B1 which served as a substrate for p38. ATM (Ataxia Telangiectasia Mutated) kinase is responsible for DDR and is defective in patients with ATM. Overexpression of lamin B1 was sufficient to induce senescence in A-T cells, demonstrating DDR-independent senescence [117]. A recent study showed that p38 MAPK collaborated with cGAS/STING and p53 pathways to induce senescence in ATM^−/−^ murine lung fibroblasts [118]. The increase in micronuclei due to increased genomic instability in ATM-deficient cells caused activation of cGAS-STING which resulted in the activation of p38 and upregulation of interferon-stimulated genes. Activation of p38 contributed to senescence via the early activation of the p53/p21 pathway, late activation of the p16 pathway and amplification of the interferon signaling, suggesting that early activation of p53/p21 was responsible for initiating cell cycle arrest whereas late activation of p16 and amplification of the interferon signaling were required for the maintenance of senescence [118].

Kwong et al. utilized molecular approaches to explore the contribution of individual p38 isoforms during senescence induction [119]. p38α, -γ and -δ but not -β were associated with Ras oncogene-induced senescence albeit with distinct mechanisms [119,126,127]. While p38α caused an increase in p16^INK4A^ mRNA, p38γ caused phosphorylation and transcriptional activation of p53, suggesting p38α and p38γ mediate Ras-induced senescence (RIS) via the p16/Rb and p53/p21 pathway, respectively [126]. In contrast, p38δ induced senescence via the p53/p21 and p16/Rb-independent pathway [119]. Oncogenic Ras induced p38δ transcription via the Raf/MEK/ERK/AP1/ETS pathway. Subsequently, p38δ was activated by MKK3/6-mediated phosphorylation causing phosphorylation of Chk1 and Chk2. Whether p38δ induced senescence via phosphorylation of Chk1 and Chk2 was not investigated.

As described in this section, senescence is regulated by multiple kinases and increase in ROS is a major mechanism in the induction of senescence phenotype. Thus, one mechanism to reverse senescence is to decrease ROS generation. It was found that combined treatment with BRAF and p38 MAPK inhibitors reduced ROS production by upregulating metallothionein 2A, improved mitochondrial function and reversed senescent phenotype [128].

### 4.5. PI3K/Akt/mTOR

Table 2 summarizes how PI3K/Akt/mTOR and AMPK signaling regulate senescence in response to different senescent inducers and under different experimental conditions.

mTOR, which acts downstream of PI3K/Akt (Figure 1), was shown to regulate SASP via several different mechanisms. When senescence was induced by oncogene, radiation, and DNA damage, mTORC1 inhibitor rapamycin suppressed secretion of proinflammatory SASP cytokines, such as IL6 and IL8, but did not restore senescence-associated cell cycle arrest in normal human fibroblasts and immortalized mammary epithelial cells [131]. The mechanism by which mTORC1 increased SASP cytokines involved increased translation of ILA1A via its downstream targets S6K1/2 and 4E-BP1 (Figure 1). IL1A, in turn, caused the activation of NF-κB which transcribes many SASP factors [140]. Secretion of proinflammatory cytokines, such as IL-6 and IL-8, from senescent cells could promote tumor growth in a paracrine manner in prostate cancer cells in vitro and in tumor xenografts [131].

Another mechanism by which mTOR regulated SASP involved translation of MK2 or MAPKAPK2 via its downstream target 4EBP1 but not p38 MAPK which acts upstream of MK2 [132]. Also in this study, inhibition of mTOR or 4EBP1 suppressed SASP but did not restore senescence-associated cell cycle arrest. MAPKAPK2 phosphorylated the RNA-binding protein ZFP36L1, reducing its activity and abundance. Since ZFP36L1 degrades cytokine mRNA, the decrease in ZFP36L1 caused an increase in the levels of SASP cytokines. mTORC1/ZFP36L1 could exert pro-tumorigenic effects in breast cancer and squamous cell carcinoma cells in a paracrine manner. mTORC1 inhibitor rapamycin suppressed tumors by inhibiting the secretion of proinflammatory SASP cytokines. It also reduced immune surveillance [132]. Thus, caution should be exercised in the use of mTOR inhibitors since they could be both beneficial and detrimental.

mTOR could also alter SASP. Therapy-induced senescence can contribute to drug resistance. Doxorubicin-induced chemoresistance in endothelial cells involved transient release of proinflammatory IL-6 due to induction of an acute stress-associated phenotype (ASAP) [134]. However, doxorubicin induced sustained release of IL6, typical of canonical SASP, in human hepatocellular carcinoma cells. The mechanism of ASAP was distinct from canonical SASP and was independent of NF-κB-mediated increase in cytokine mRNA but involved increased translation of IL-6 by p38 MAPK which was activated by doxorubicin-induced generation of ROS. The lack of typical SASP in endothelial cells was due to a decline in PI3K/Akt/mTOR activity during the acquisition of senescence. Restoring PI3K/Akt/mTOR activity switched ASAP to SASP, resulting in a sustained release of SASP factors. While doxorubicin-treated senescent endothelial cells did not exhibit typical enlarged morphology, activation of PI3K/Akt/mTOR restored enlarged morphology [134]. Thus, mTOR inhibitors could prevent chronic inflammation and maintain tissue homeostasis by maintaining ASAP.

A new type of senescence was introduced by Pandolfi and colleagues and was named Pten-loss-induced cellular senescence (PICS) [13]. It is dependent on the stabilization of p53/p21 via upregulation of p19^ARF^. Loss or mutation of PTEN is frequent in prostate cancers. PICS suppressed tumorigenesis in PTEN-deficient prostate and loss of both PTEN and p53 caused prostate cancer invasion [129]. PICS was distinct from oncogene-induced senescence since it could be triggered rapidly in the absence of DDR. Since PTEN negatively regulates PI3K (Figure 1), loss or inactivation of PTEN causes activation of the PI3K/Akt/mTORC1 pathway. PICS involved not only increased stabilization of p53 but also increased translation of p53 by mTORC1 and suppressed prostate tumor growth in vivo. PTEN KD also induced senescence via p53/p21-dependent DDR-independent pathway via an increase in p53 stabilization in MCF-7 breast cancer cells [130]. In the absence of PTEN, both mTORC1 and mTORC2 associated with p53 in competition with MDM2, phosphorylated p53 at Ser15 and induced senescence via the p53/p21 pathway. PTEN KD-induced senescence could be restored by the inhibitors of PI3K/Akt and mTORC1/2 but not by the inhibitors of ERK1/2, p38 MAPK or JNK.

Similar to PICS, hyperactivation of Akt isoforms CA-AKT-, PIK3CA^E545^ mutant PI3K- (associated with several cancers) or PTEN KD-induced senescence in immortalized human fibroblasts via p53-dependent, DDR-independent mechanism [12]. Akt-induced senescence (AIS) also involved mTORC1-dependent translation of p53 and increased stabilization of p53 by Akt-mediated phosphorylation of MDM2 but did not involve phosphorylation of p53 at Ser15 or stabilization of p53 by p19^ARF^. Additionally, AIS was independent of p16 and SAHF formation and induced a SASP program that was distinct from OIS-induced SASP [12]. These studies question the appropriateness of targeting mTORC1 in cancers with hyperactive PI3K/Akt/mTORC1 since suppression of senescence by mTORC1 inhibitors could promote tumorigenesis.

To identify unique mediators of Akt-induced senescence, a combination of transcriptomic and metabolic profiling was used to create a signaling network in *hTERT*-immortalized BJ foreskin fibroblasts [141]. There was considerable metabolic rewiring during both oncogene-induced senescence (OIS) and Akt-induced senescence (AIS). Interestingly, while activation of Ras/ERK signaling induced OIS, inhibition of Ras/ERK was required for the maintenance of AIS. Neurofibormin 1 (NF1), which stimulates GTPase activity of GTP-bound Ras to convert active Ras to its inactive form, was responsible for the suppression of Ras/ERK signaling via a negative feedback loop. NF1 deficiency could promote transformation by reversing AIS whereas inhibition of MEK/ERK signaling could induce senescence in cells with hyperactive PI3K/Akt/mTOR. Thus, it is essential to consider the crosstalk and feedback regulation among signaling pathways prior to targeting any particular pathway.

Global transcriptomic analysis identified the involvement of mitochondria in the development of SASP [133]. During DNA damage-induced senescence, activation of mTORC1 integrated PGC-1β (peroxisome proliferator-activated receptor gamma coactivator 1β)-dependent mitochondrial biogenesis with ROS-driven DDR both in vitro and in vivo. Mitochondria were involved in both the development and maintenance of senescence. Depletion of mitochondria decreased mTORC1 activity and reversed some of the features of senescence, such as SASP [133]. As observed previously [131,132], mTOR inhibition suppressed SASP but did not restore cell cycle arrest. The authors suggested that mitochondria could provide as a therapeutic target to limit inflammation during aging.

Circulating tumor cells (CTC) are required for the dissemination of a primary tumor to a distant site during metastasis [142]. The cytoskeletal regulator cortactin encoded by CTTN has been associated with cancer progression and metastasis [143]. A recent study showed that CTCs that are low in CTTN exist in a pre-senescent state contributing to therapy resistance and tumor progression [135]. The mechanism by which depletion of CTTN contributed to senescence in melanoma CTCs involved hyperactivation of mTORC1 in Rab7-positive late endosomes causing mTORC1-mediated phosphorylation of p53 at S15 and S33. Activation of p53 induced p21 causing inhibition of CDK1. Since DRP1 (dynamic-related protein 1), a substrate for CDK1, mediates mitochondrial fission, inhibition of DRP1 resulted in mitochondrial dysfunction causing increased mitochondrial ROS production which further activated p53. This p53-CDK1-DRP1-mtROS-p53 positive feedback loop was essential to maintain stable senescence in CTCs and was associated with therapy resistance [135]. In fact, when CTCs were isolated from melanoma patients undergoing treatment, senescent CTCs were associated with disease progression and therapy resistance. A two-step strategy that combined induction of senescence by knocking down cortactin followed by the elimination of senescent cells with a senolytic was successful in reducing tumor growth and lung metastases in melanoma xenograft.

### 4.6. AMPK

AMP-activated protein kinase (AMPK) is activated during energy crisis. Cell cycle progression requires energy, and glucose serves as the energy source. When mouse embryo fibroblasts (MEFs) were grown in low glucose, AMPK became activated and induced premature senescence by phosphorylating p53 at Ser15 resulting in its activation [46]. Although DNA damage also induces phosphorylation of p53 at the same site, AMPK-mediated p53 phosphorylation and cell cycle arrest was independent of DNA damage signaling. It was suggested that metabolic activation of p53 was used to conserve available glucose to maintain the viability of senescent cells.

Activation of AMPK was also associated with oncogene-induced senescence. Oxidative DNA damage by oncogenic H-Ras caused p53- and p16/Rb-mediated mitochondrial dysfunction which led to a drop in cellular ATP causing activation of AMPK, increased ROS production and induction of senescence [137]. In this study, whether activation of AMPK was a consequence of mitochondrial dysfunction and senescence induction or activation of p53 by AMPK was responsible for senescence induction was not investigated.

Campisi and colleagues provided direct evidence that mitochondrial dysfunction-associated senescence, which they termed MiDAS, was mediated by the activation of AMPK and was independent of DNA damage and ROS [138]. Mitochondrial dysfunction caused a drop in the NAD^+^/NADH ratio, resulting in the activation of AMPK which induced senescence via phosphorylation and activation of p53. MiDAS induced atypical SASP and was associated with the secretion of IL-10, TNF-α and CCL27, but IL-1 secretion was suppressed due to inhibition of NF-κB by p53. MiDAS also occurred in vivo and MiDAS SASP could inhibit adipogenesis and promote keratinocyte differentiation [138].

N-methyl-N-nitrosourea (MNU)-induced DNA damage caused retinal degeneration via generation of ROS which caused activation of AMPK and induction of senescence in 661w cells derived from retinal tumors [139]. While the authors speculated that AMPK induced senescence via ULK1, it is not clear if the activation of ULK1 which is a substrate for AMPK is the cause or consequence of AMPK-mediated induction of senescence.

Activation of AMPK was also associated with a decrease in senescence [144]. Type 2 diabetes mellitus (T2DM) is associated with β-cell senescence which contributes to aging. Increase in glucagon during exercise caused activation of AMPK in pancreatic islets, resulting in nuclear translocation of NRF2 and decreased transcription of senescence markers p21 and p16^Ink4A^.

## 5. Coordinate Regulation of Autophagy and Senescence by Kinases

This section focuses on the mechanistic information from selected articles to explore how kinases decide cell fate by regulating both autophagy and senescence. Since there is crosstalk among kinase signaling pathways, a clear-cut separation about the involvement of a particular kinase may not be feasible.

### 5.1. PI3K/Akt/mTOR

**Figure 2:**
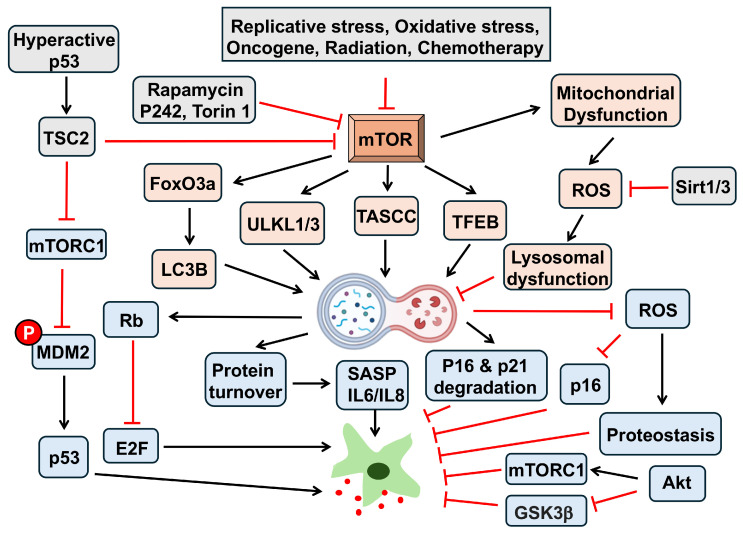
Dual regulation of autophagy and senescence by mTOR. See text for details. The autophagy icon is from Biorender.com.

The seminal observation by Young et al. that a decrease in mTORC1 and mTORC2 activity during Ras oncogene-, CA-MEK- and DNA damage-induced senescence in human diploid fibroblasts (IMR90) was associated with an increase in autophagy first suggested a link between autophagy and senescence via mTOR, the master regulator of autophagy [11]. Although the PI3K/Akt/mTOR pathway acts downstream of Ras, mTORC1/2 activity declined following a transient activation due to a negative feedback loop between mTORC1 target S6K1 and PI3K/Akt (Figure 1) and the decrease in mTOR activity correlated with the induction of senescence [39]. Both mTORC1 and mTORC2 cooperated during senescence induction. Inhibition of mTORC1 activated autophagy via ULK1/3 and overexpression of ULK3 induced both autophagy and senescence (Figure 2). Inhibition of mTORC2 was associated with FoxO3a-mediated increase in autophagy-related genes, such as LC3B (Figure 2). Inhibition of autophagy delayed the production of SASP cytokines IL-6 and IL-8 but failed to reverse senescence-associated cell cycle arrest. Thus, activation of autophagy during senescence induction was required for the establishment of senescence. This requirement for autophagy for senescence establishment was further supported by in vivo studies with mouse papilloma harboring mutant Ras. The authors speculated that rapid protein turnover during oncogene-induced senescence (OIS) facilitated the establishment of senescence.

In a subsequent study, Narita et al. [145] provided evidence that protein degradation during autophagy served an increased demand for protein translation during senescence. During OIS, mTOR accumulated in a cellular compartment named TOR-autophagy spatial coupling compartment (TASCC) which was enriched in autolysosomes as well as SASP factors and excluded autophagosomes that are formed during starvation-induced autophagy (Figure 2). Disruption of mTORC1 recruitment to TASCC inhibited IL-6/IL-8 synthesis. Thus, autophagy-mediated protein degradation could generate amino acids to support the synthesis of SASP components within the same compartment. In these studies, the direct involvement of mTORC1 and mTORC2 was not investigated. However, the formation of TASCC strongly supports the involvement of mTORC1 in SASP cytokine production.

Both PI3K/Akt pathway and Ras are oncogenic, but unlike the Ras oncogene which induced both senescence and autophagy, activation of PI3K/Akt failed to induce autophagy or complete the senescence program [146]. However, activated Akt1 antagonized the effects of oncogenic Ras on the induction of autophagy and senescence. Akt activates mTORC1 which inhibits autophagy whereas Akt phosphorylates and inhibits its substrate GSK3β which is involved during Ras-induced senescence. Thus, restoration of mTOR activity and inhibition of GSK3β by Akt contributed to the antagonism (Figure 2). Suppression of Ras-induced senescence by activated PI3K/Akt facilitated the development of pancreatic intraepithelial neoplasms to pancreatic ductal adenocarcinoma in vivo. This study provides an explanation why activating mutation in both Ras and PI3K/Akt is found in many cancers, such as pancreatic and colon cancers. Since inhibition of mTORC1 by rapamycin could reactivate senescence in tumors with activating mutations in both the Ras and PI3K/Akt pathway, targeting mTORC1 could provide a therapeutic opportunity for these tumors.

Several cancer cells, including head and neck and glioblastoma cells, activated autophagy to protect against radiation-induced DNA damage. Combined treatment with radiation and mTOR inhibitors prolonged autophagy and enhanced rather than protected against radiosensitivity both in vitro and in vivo [147]. While DNA damage induced transient increase in autophagy and reversible cell cycle arrest via induction of p53/p21, the mTOR inhibitor prolonged autophagic flux and induced senescence via activation of Rb. Since Rb binds to E2F and represses transcription of E2F target genes that are required for cell cycle progression, activation of Rb induced cell cycle arrest by inhibiting E2F (Figure 2). Thus, combining mTOR inhibitors with radiation could be effective in treating radioresistant tumors. In this study, PP242, which inhibits both mTORC1 and mTORC2, was more effective than rapamycin which primarily inhibits mTORC1, suggesting possible involvement of both mTORC1 and mTORC2 in augmenting autophagy and senescence in response to radiation.

Activation of autophagy was also needed for radiation-induced senescence and protected against radiation-induced cell death in breast cancer cells depleted of pituitary tumor-transforming 1 (PTTG1/securin), an oncogene which contributes to tumor invasion and metastasis [148]. Inhibition of autophagy sensitized these cells to radiation by switching senescence to apoptosis. Autophagy was required for the release of SASP factors, such as colony-stimulating factor 2 (CSF2) which caused activation of JAK2-STAT3 and AKT to promote migration and invasion in unirradiated neighboring cells but inhibited endogenous autophagy. Inhibition of JAK2 or mTOR could rescue endogenous autophagy which in turn antagonized radiation-induced bystander effects on invasion and metastasis. This study illustrates how JAK2-STAT3 and AKT-mTOR signaling orchestrates autophagy and senescence to decide the fate of senescent cancer cells and neighboring cells.

Autophagy was also positively correlated with senescence when glioblastoma U87 cells were treated with the anticancer drug temozolomide (TMZ) which caused transient activation of AMPK/ULK1 and p38 MAPK and prolonged inhibition of the PI3K/Akt/mTOR pathway. Activation of autophagy by the mTORC1 inhibitor rapamycin potentiated TMZ-induced senescence [149]. Thus, in a population of cells, autophagy was positively correlated with senescence. However, the causality between autophagy and senescence was challenged when glioma cells treated with temozolomide (TMZ) were analyzed at the single-cell level. There was no correlation between the activation of autophagy and induction or decline of senescence, suggesting senescence occurred independent of autophagy.

In contrast to the above studies, inhibition of autophagic flux accelerated senescence in primary human fibroblasts obtained from young healthy donors and was referred to as autophagy impairment-induced senescence (AIPS) [150]. The characteristics of AIPS resembled replicative senescence and were associated with a decrease in mTOR activity and protein synthesis. Since autophagy is responsible for the clearance of damaged mitochondria, inhibition of autophagy led to the accumulation of dysfunctional mitochondria, causing increased ROS production and activation of p53/p21 which were responsible for the induction of AIPS. However, ROS scavenger or p53 inhibitor not only decreased markers of senescence but also restored mTORC1 activity which inhibits autophagy, suggesting a positive association between the two. During AIPS, a decrease in mTOR activity was a late event and was followed by the prolonged increase in mTORC1 activity. Since autophagic flux generates amino acids which activate mTORC1, it is conceivable that reduction in mTORC1 activity was a consequence of a decreased generation of amino acids due to the inhibition of autophagic flux. Whether inhibition of mTORC1 was required for the induction or maintenance of senescence during AIPS was not investigated.

Impairment of autophagic flux was also associated with oxidative stress-induced senescence in NIH3T3 and MRC5 cells [151]. Since lysosomal function is required for the degradation of cargo and completion of the autophagic process, dysfunctional lysosomes contributed to the impairment of autophagic flux. Mitochondrial dysfunction and ROS overproduction occurred prior to lysosomal dysfunction which was responsible for the impairment of autophagic flux and development of senescence (Figure 2). mTOR inhibitors (rapamycin and PP242) improved mitochondrial and lysosomal functions and attenuated senescence by restoring autophagic flux. However, only mTOR-dependent autophagy activators, but not other autophagy activators such as valproic acid or LiCl, exerted an anti-senescence effect. The authors suggested that the modulation of autophagy at the late stage is more effective compared to early-stage modulation for preventing senescence.

Inhibition of mTOR also restored autophagic flux during doxorubicin-induced DNA damage and suppressed senescence. The mechanism by which mTORC1 regulated doxorubicin-induced autophagy and senescence in lung cancer A549 cells involved Sirt3, a mitochondrial deacetylase and an antioxidant [152]. Doxorubicin increased the formation of autophagosomes, but autophagic flux was blocked due to the activation of the PI3K/Akt/mTOR pathway. Sirt3 restored the impairment of autophagic flux by inhibiting doxorubicin-induced activation of PI3K/AKT/mTOR and by scavenging ROS generated by doxorubicin (Figure 2). A similar PI3K/AKT/mTOR-mediated anti-senescence function of Sirt1-mediated autophagy was also reported in doxorubicin-treated MCF-7 cells by the same group [153].

mTORC1 also played an important role in regulating the fitness of skeletal muscle stem cells or satellite cells. The regenerative capacity of skeletal muscle stem cells is preserved during the quiescent state and is declined during aging. Autophagy removes damaged organelles and proteins to maintain organelle integrity and protein homeostasis (proteostasis). It was found that high basal autophagy/mitophagy maintained the quiescence state of mouse satellite cells derived from young mice and was impaired in satellite cells from old mice [154]. Decline in mitophagy caused an accumulation of damaged mitochondria and increased ROS production which caused induction of p16^INK4A^ by the epigenetic mechanism and cells entered senescence. Genetic impairment of autophagy also induced senescence in young cells whereas treatment of old mice with mTORC1 inhibitor rapamycin restored autophagy and suppressed senescence. Rapamycin improved mitochondrial function, decreased ROS, suppressed senescence and restored the quiescent state by inducing mitophagy. Similar results were found in aged human cells, suggesting a causal role of autophagy impairment in senescence and a potential use of mTORC1 inhibitors to slow the aging process.

mTORC1 activity was also affected during the acquisition of senescence. When senescence was induced in human fibroblasts by oncogene, replication exhaustion or irradiation, mTORC1 activity persisted and remained insensitive to nutrients and external stimuli [155]. This was due to depolarization of the plasma membrane in senescent cells, causing activation of PI3K/Akt which acts upstream of mTORC1. In addition, an increase in basal autophagy in senescent cells generated free amino acids which also caused activation of mTORC1. Inhibition of mTORC1 by Torin 1 or Akt augmented autophagy and induced non-apoptotic cell death only in senescent cells and in the absence of amino acids or serum, suggesting hyperactivation of autophagy may result in cell death. Thus, persistent mTORC1 activity supported the survival of senescent cells. Since Torin 1 inhibits both mTORC1 and mTORC2 and Akt acts downstream of mTORC2, it is conceivable that mTORC2 contributed to the survival of senescent cells by preventing autophagic cell death.

The direct involvement of mTORC2 in the interplay between starvation-induced autophagy and senescence was demonstrated in WI-38 human embryonic lung fibroblasts [156]. Sustained autophagy by prolonged starvation increased ROS production causing activation of mTORC2 and induction of senescence which was inhibited by silencing of Atg7 or blockage of autophagosome formation by the PI3K inhibitor 3-methyl adenine (3-MA). While inhibition of mTORC2 by rapamycin or rictor knockdown inhibited p16, p21 and SA-β-gal activity, inhibition of mTORC1 by raptor knockdown attenuated only SA-β-gal activity but had little effect on p16 and p21 levels, suggesting that mTORC1 and mTORC2 acted via distinct mechanisms and mTORC2 was primarily involved in senescence induction during prolonged serum starvation.

SP0495 protein encoded by the open reading frame of KIAA0495 is a phosphoinositide binding protein which inhibits Akt and its downstream targets, including mTOR and NF-κB [157]. It was shown to function as a tumor suppressor both in vitro and in vivo by inducing senescence via the p53/p21 pathway and autophagy via stabilization of beclin 1 involving inhibition of PI3K/Akt/mTOR. In this study, however, the causality between autophagy and senescence was not investigated.

A systems biology approach was used to understand how multiple signaling pathways interact to drive senescence so that strategies could be developed to limit senescence progression. A comprehensive dynamic model of irradiation-induced senescence combined with in silico prediction and in vitro validation identified two interventions [158]. First, scavenging of ROS and inhibition of mTOR could restore mitochondrial health and delay senescence by reducing DNA damage caused by mitochondrial dysfunction. Second, activation of AMPK and mitophagy could control cellular energy status. Combined interventions were more effective than a single intervention in improving mitochondrial function but could not restore the pre-senescent state. Since mitophagy only eliminated new but not old mitochondria, mTORC1-dependent mitochondrial biogenesis and accumulation of old mitochondria caused persistent mitochondrial dysfunction and cells entered a new senescent state. Thus, interventions were more effective at early stages to prevent senescence progression and inflammation and gradually declined with time. This study provides useful information regarding the dynamics of mitophagy and senescence but did not include paracrine effects of SASP. In addition, there is significant crosstalk among kinase signaling pathways. Systems biology approaches that integrate how multiple kinase pathways interact as a network to coordinate both processes need to be explored.

Using an integrated approach that combined quantitative proteomics and protein stability, Lee et al. [28] developed senescence-associated autophagy interactome (SAAI) to identify autophagy substrates during stress-induced senescence. The authors demonstrated that selective autophagy of several proteins serves distinct functions during the development of senescence. For example, p62-mediated selective degradation of KEAP1 (Kelch-like ECH-associated protein 1) maintained viability of senescent cells by sustaining redox homeostasis via an increase in the antioxidant Nrf2 but did not affect SA-β-gal activity or IL1A expression. On the other hand, OPTN-mediated selective autophagy of TNIP1, a negative regulator of NF-κB, enhanced inflammation via sustained activation of SASP whereas NDP52-mediated selective degradation of eIF3 proteins supported long-term viability of senescent cells by maintaining proteostasis. This selective autophagy network also appears to be operated in vivo during progression of human osteoarthritis. The selective degradation of these proteins was mediated by the inhibition of ULK1 and not by the inhibition of mTORC1 which induced bulk autophagy. Whether ULK1 was regulated by AMPK, p38 MAPK or some other kinase was not investigated. This study provided important insights regarding how selective autophagy regulates different steps of senescence to acquire the final stage of senescence during DNA damage-induced senescence and emphasizes the importance of proteostasis in SASP development. Similar network analyses should be extended to different senescent inducers and disease models.

mTORC1 could also regulate senescence via selective autophagy of senescent inducers p16 and p21 [159]. During age-dependent degenerative heart valve disorder, transition of quiescent valve interstitial cells (qVICs) to senescent myofibroblasts (aVICs) was associated with activation of PI3K/Akt/mTOR signaling and impairment of autophagic flux. Activation of autophagy by mTOR inhibitor rapamycin attenuated senescence by decreasing the p16 and p21 protein but not mRNA. The mechanism by which p16 and p21 protein levels decreased involved the autophagy adaptor p62 which sequestered p16 and p21 in autolysosomes where they were degraded by lysosomal hydrolases. Inhibition of autophagy caused upregulation of p16 and p21 by preventing their degradation via selective autophagy. Thus, selective protein degradation could be exploited to treat age-related degenerative diseases.

A recent study demonstrated the involvement of mTORC1 acting downstream of p53/TSC2 in regulating both autophagy and senescence in lung adenocarcinoma [160]. *KRAS* is frequently mutated in lung and pancreatic cancers. Restoration of p53 activity especially with the hyperactive p53^53,54^ transactivation mutant promoted tumor regression via transcriptional activation of TSC2 which negatively regulates mTORC1. The transcription factor EB (TFEB), a substrate for mTORC1, is involved in the transcription of genes required for lysosome biogenesis and autophagy [161]. Inhibition of mTORC1 induced nuclear translocation of TFEB which stimulated the expression of lysosomal genes causing an induction of autophagy (Figure 2). Inhibition of mTORC1 by TSC2 also prevented MDM2-mediated proteosomal degradation of p53. Since MDM2 is a substrate for mTORC1 which phosphorylates and activates MDM2 ubiquitin ligase, inhibition of mTORC1 inhibited MDM2-mediated degradation p53 and triggered senescence via p53/p21 (Figure 2). The MDM2 inhibitor also induced autophagy followed by senescence via the TSC2/mTORC1/TFEB pathway in vivo. Secretion of SASP cytokines, such as CCL3, CCL4 and MIP1 from senescent cells, recruited macrophages to clear senescent lung cancer cells causing regression of lung adenocarcinoma. Thus, this study not only substantiates the importance of mTOR signaling in regulating autophagy and senescence but also provides a mechanistic-based therapeutic strategy to treat lung cancer.

### 5.2. AMPK/ULK1

**Figure 3:**
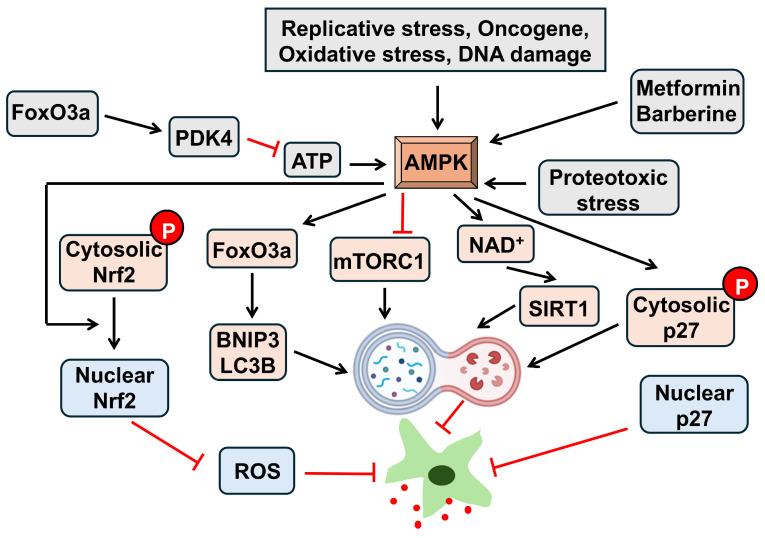
Dual regulation of autophagy and senescence by AMPK. See text for details. The autophagy icon is from Biorender.com.

AMPK activity, which maintains cellular energy homeostasis, is declined with aging [162]. When senescence was induced in human embryonic lung fibroblasts by oxidative stress or Ras oncogene, AMPK was activated via the FoxO3a transcription factor. The mechanism by which FoxO3a activated AMPK involved transcriptional activation of the mitochondrial enzyme pyruvate dehydrogenase kinase 4 (PDK4) which decreased the cellular ATP level, causing an increase in AMPK activity which in turn induced autophagy through inhibition of mTORC1 (Figure 3) [163]. Inhibition of autophagosome formation by 3-MA and knockdown of FoxO3a decreased senescence and induced apoptosis. The authors concluded that increased autophagy by AMPK activation/mTORC1 inhibition was required for the induction of senescence. In this study, autophagic flux was not determined, and direct involvement of AMPK was not assessed.

Oncogenic Ras (RASv12) or c-Myc also activated AMPK and induced autophagy in mammary epithelial MCF-10A or MCF-7 breast cancer cells. Mitochondrial stress augmented oncogenic Ras-transformed but not non-transformed cells to autophagy and inhibited cell growth in vitro and in an MCF-7-RASv12 tumor xenograft [164]. Mitophagy clears damaged mitochondria and maintains cellular energy status. Combination of oncogenic stress with mitochondrial stress caused an overactivation of AMPK which activated the FoxO3 transcription factor causing upregulation of its transcriptional targets BNIP3 and LC3B involved in mitophagy/autophagy (Figure 3). Inhibition of either AMPK or FoxO3 partially restored cell growth, suggesting AMPK-FoxO3-mediated autophagy suppressed cell growth. While activation of oncogenes is known to induce senescence to suppress tumorigenesis and overactivation of autophagy can induce autophagic cell death, this study did not discern whether inhibition of cell growth or tumor suppression involved apoptosis, senescence or autophagy.

Activation of AMPK also promoted senescence in B-RAF-dependent melanoma cells that express the plasma-membrane potassium channel Kv11.3 (hERG3) [165]. Stimulation of hERG3 with a small molecule activator NS1643 induced autophagic flux via the AMPK/ULK1 pathway followed by the induction of senescence. Inhibition of AMPK in NS1643-treated cells induced apoptosis, suggesting that AMPK promoted cell survival by inducing autophagy and acquisition of the senescent phenotype. In this study, the causality between autophagy and senescence was not investigated.

Anti-mitotic drugs, such as paclitaxel, are used for the treatment of many cancers but slippage of mitotic arrested cells could cause drug resistance. The proteotoxic stress of the endoplasmic reticulum in tetraploid postslippage U2OS, HCT116 and hTERT immortalized RPE-1 cells caused activation of AMPK and induction of autophagy via the AMPK/ULK1/mTORC1 pathway and cells entered a senescent state [166]. Release of proinflammatory SASP cytokines from senescent cells exerted paracrine tumorigenic effects. Inhibition of autophagy could bypass senescence and suppress pro-tumorigenic effects of SASP by inducing p53-dependent apoptosis. Thus, a combination of antimitotic chemotherapeutic drugs with autophagy inhibitors could provide a strategy to combat drug resistance.

Activation of autophagy by AMPK could also protect against oxidative stress-induced senescence. As has been observed during aging, AMPK activity also declined when senescence was induced by oxidative stress in murine and human fibroblasts and HUVEC cells and affected lysosomal function in senescent cells independent of the mTORC1/TFEB pathway [167]. The mechanism by which AMPK coordinated autophagy and senescence involved NAD^+^ biosynthesis which was downregulated in senescent cells. Like ATP, NAD^+^ is required for maintaining cellular energy. In addition, NAD^+^ is a cofactor of the Sirt family deacetylases [152]. Activation of AMPK attenuated senescence by restoring NAD^+^ homeostasis and activating autophagic flux via Sirt1 (Figure 3).

Activation of AMPK by metformin also alleviated oxidative stress-induced senescence in human lens epithelial cells by restoring autophagic flux. Oxidative damage contributes to age-related cataract (ARC). Patients with ARC exhibited an increase in senescence markers compared to age-matched controls [168]. AMPK activity declined in ARC patients and during oxidative stress-induced senescence of human lens epithelial cells. The mechanism by which inhibition of AMPK contributed to an increase in senescence involved lysosomal dysfunction which led to the impairment of autophagic flux and induction of senescence. The anti-senescence effect of metformin was validated using an aged mouse model [169]. Since inhibition of AMPK is associated with ARC, activation of AMPK by FDA-approved metformin could be exploited for the treatment of patients with ARC.

Activation of AMPK also induced autophagy and inhibited senescence in geriatric muscle stem cells (MuSc). Autophagy declined progressively in MuScs isolated from young, middle-aged and old mice and was associated with a concomitant decline in AMPK activity and an increase in apoptosis [170,171]. Geriatric MuScs also exhibited an increase in senescence which was attenuated by the activation of AMPK. The cell fate decision by AMPK was mediated via CDK inhibitor p27, a substrate for AMPK (Figure 3). While cytoplasmic p27 was associated with cell proliferation and autophagy, nuclear localization of p27 was associated with apoptosis and senescence. Phosphorylation of p27 at Thr198 by AMPK declined during aging, causing its nuclear localization. Activation of AMPK could suppress senescence by causing cytoplasmic translocation of p27 (Figure 3). Thus, inhibition of AMPK-mediated autophagy resulted in apoptosis and induction of senescence in geriatric cells.

The transcription factor Nrf2 is also a substrate for AMPK (Figure 3) [172]. Downregulation of casein kinase 2 (CK2) induced senescence in MCF-7 and HCT116 cells via Nrf2 [173]. CK2 caused activation of AMPK which phosphorylated Nrf2 and increased Nrf2 activity by inducing its nuclear translocation (Figure 3). CK2 also stabilized Nrf2 by autophagy-mediated degradation of Keap1. Thus, downregulation of CK2 reduced the Nrf2 level via Keap1-mediated degradation, and attenuated AMPK-mediated nuclear import of Nrf2. Since Nrf2 maintains redox homeostasis, a decrease in Nrf2 level/activity increased the generation of ROS and induced cellular senescence [173]. Thus, cellular localization of AMPK substrates played an important role in regulating autophagy and senescence.

Autophagy not only regulates senescence but can also alter SASP both qualitatively and quantitatively. The anticancer drug temozolomide (TMZ), a drug of choice for the treatment of brain cancers, induced senescence in glioblastoma cells. Inhibition of autophagy initiation by ULK1 knockdown or blockage of autophagy by Atg3 or Atg5 knockout augmented TMZ-induced senescence as well as its anticancer activity in glioblastoma cells and tumor xenografts [174]. The mechanisms by which autophagy inhibition by ULK1 enhanced the tumor-suppressive effect of TMZ involved modification of SASP factors which affected the tumor microenvironment in a paracrine fashion. The altered SASP components increased immune surveillance by recruiting M1 macrophages and decreased polarization of immune-suppressive M2 macrophages. Inhibition of autophagy in combination with TMZ also enhanced antitumor immunity in vivo by reducing recruitment of neutrophils. This study provides important mechanistic insight regarding how inhibition of autophagy combined with chemotherapeutic drug-induced senescence could be exploited to improve cancer therapy by enhancing anti-tumor immunity.

Activation of AMPK could also promote diabetic wound healing by suppressing senescence [175]. One of the detrimental effects of excessive accumulation of senescent cells is delayed wound healing. In a type 1 diabetes mouse model, activation of AMPK by a pharmacologic inhibitor A769662 accelerated wound healing by eliminating senescent cells near the wound site. The mechanism by which AMPK removed senescent cells involved induction of ferroptosis or iron-dependent cell death by ferritinophagy, a type of selective autophagy. Iron metabolism is regulated by ferritin. Nuclear receptor coactivator-4 (NCOA4) can bind to and target ferritin to lysosome where it is degraded by autophagy/ferritinophagy. This causes release of iron from the iron store and induction of ferroptosis. Activation of AMPK induced NCOA4-dependent ferritinophagy to eliminate senescent cells via ferroptosis. Thus, AMPK activators could promote would healing by removing senescent cells.

### 5.3. MEK/ERK

Autophagy and senescence are regulated by the crosstalk among multiple signaling pathways. In A549 lung adenocarcinoma cells harboring mutated K-Ras or rodent fibroblasts transformed with *E1A* and *cHa-Ras* oncogenes (ERas cells), inhibition of MEK/ERK induced mTORC1-independent AMPK-dependent cytoprotective autophagy/mitophagy which removed damaged mitochondria to allow the survival of MEK/ERK inhibitor-treated cells [176]. However, when senescence was induced by the treatment with an HDAC inhibitor, MEK/ERK inhibitor-treated cells underwent caspase 8-mediated apoptosis. Although AMPK was activated by the MEK/ERK inhibitor in senescent cells, the formation of autolysosomes was impaired because LAMP1 and mTORC1 localized in the perinuclear region instead of lysosomes and Ras redistributed from the plasma membrane to the cytosol where it associated with LC3. The spatial separation of autophagosomes and lysosomes caused accumulation of damaged mitochondria, increased production of ROS and decreased glycolysis resulting in apoptotic cell death. Thus, combining ERK inhibitors with senescent inducers could suppress tumors with mutant Ras.

Since mTORC1 remained active in MEK/ERK inhibitor-treated cells, the same group examined the effects of the mTOR inhibitor pp242 on cell fate [177]. Low concentrations of pp242 but not rapamycin caused a transient increase in mitochondrial damage, ROS production and autophagic flux followed by the inhibition of autophagy in senescent ERas cells. The mTOR inhibitor failed to induce cell death in senescent cells since the damaged mitochondria were eliminated in special LC3-lacking vacuoles containing lysosomes without completion of the autophagy process. However, when MEK/ERK was inhibited, pp242 failed to restore AMPK-mediated autophagy or segregate damaged mitochondria and therefore was unable to restore viability of senescent cells. Thus, MEK/ERK activity appears to be required for the formation of special LC3-lacking vacuoles. One important outcome from this study was that spatial organization of senescent cells rather than mTORC1 activity dictated cell fate. Since pp242 inhibits both mTORC1 and mTORC2 whereas rapamycin at low concentrations primarily inhibits mTORC1, a lack of the ability of rapamycin to induce mitochondrial damage suggests possible involvement of mTORC2.

Silencing of *KRAS* or inhibition of ERK1/2 also enhanced autophagic flux in several pancreatic ductal adenocarcinoma cells containing mutant KRAS and in KRAS-driven mouse model of pancreatic cancer. The mechanism by which the ERK1/2 inhibitor increased autophagic flux involved activation of AMPK, decreased glycolysis and impaired mitochondrial function [178]. Combined inhibition of ERK signaling and autophagy induced cell death in PDAC cells, inhibited growth of the PDAC organoid and exhibited anti-tumor activity in patient-derived xenograft models. In this study, whether the ERK inhibitor or a combination of ERK and the autophagy inhibitor affected senescence was not investigated.

The involvement of ERK1/2 in autophagy and senescence during pancreatic cancer development was provided by Yan et al. [179]. Interaction between cancer cells and stromal cells is required for cancer progression. High ERK1/2 activity in cancer-associated pancreatic stellate cells (PSC) present in the tumor microenvironment promoted interaction between pancreatic cancer cells (PCC) and PSCs and enhanced migration and invasion of PCCs contributing to highly metastatic pancreatic cancer. Inhibition of ERK1/2 suppressed PCC migration and invasion by interfering with PCC-PSC interaction and this was associated with an increase in autophagy and upregulation of senescence marker p16 but SASP cytokine IL6 and matrix metalloproteases were decreased, suggesting that the ERK1/2 inhibitor may have suppressed the paracrine effect of SASP on tumor migration and invasion although the effect of ERK1/2 inhibitor on SASP was not monitored. Inhibition of ERK inhibitor-induced autophagy triggered senescence in PSCs but apoptosis in PCCs. The ERK inhibitor also synergized with the autophagy inhibitor chloroquine to suppress liver metastasis in vivo. Thus, combining ERK inhibitors with autophagy inhibitors could be useful in treating metastatic pancreatic cancer. The mechanism by which the ERK1/2 inhibitor regulated autophagy and senescence was not explored.

### 5.4. p38 MAPK

p38 MAPK is activated by cellular stress and was shown to play a causal role in senescence [111]. Several studies showed that the activation of p38 also induces autophagy [70,76] and both processes may be interdependent. In human diploid WI-38 fibroblasts, oxidative stress induced autophagy and senescence via increased production of ROS and activation of p38 MAPKα [180]. p38 activated autophagy via an increase in beclin-1, ULK3 and LC3B mRNA and induced senescence via the upregulation of p21 at the posttranscriptional level and not via p53-mediated transcriptional activation. Inhibition of autophagy by p38 MAPK inhibitor SB203580 attenuated oxidative stress-induced senescence by downregulating p21, suggesting that autophagy and senescence may be causally linked via p21. The mechanism by which p38 MAPK induced or stabilized p21 was not determined.

Plant-derived Rhus coriaria ethanolic extract (RCE) also induced both autophagy and senescence in breast cancer MDA-MB-231, MCF-7 and T47D cells via the activation of both p38 and ERK1/2 [181]. RCE-induced DNA damage caused activation of p38 and an induction of autophagy which preceded senescence. Inhibition of autophagy attenuated senescence, suggesting that autophagy was responsible for the induction of senescence. Blockage of both autophagosome and autolysosome formation inhibited p38 activity whereas only autolysosome maturation inhibited ERK1/2. The authors speculated that p38 was responsible for both the initial and final stages of autophagy whereas ERK1/2 interfered only with autolysosome maturation. In this study, activation of p38 may be the consequence of autophagy induction rather than a mediator of autophagy. Direct involvement of p38 and ERK1/2 on RCE-mediated autophagy and senescence was not examined.

Slobodnyuk et al. used a combination of p38-selective inhibitor BIRB 796 and genetic manipulation of p38 to provide convincing evidence regarding the involvement of p38 in autophagy and senescence [182]. p38α activation was sufficient to induce autophagic flux in osteosarcoma cells. The mechanism of p38α-mediated autophagy involved direct phosphorylation of ULK1 at the Ser555 site and increased mitochondrial ROS production via its downstream target MK2 (MAPK-activated protein kinase 2). Sustained p38α activation also induced senescence via upregulation of p21 mRNA independent of p53. These results corroborate the findings of Luo et al. in human fibroblasts [180]. Activation of autophagy by p38 protected cells against chemotherapeutic drug-induced apoptosis by inducing senescence. Thus, p38 inhibitors could sensitize cancer cells to chemotherapeutic drugs.

Activation of p38 was also associated with a decrease in autophagy. When senescence was induced by replication exhaustion in human diploid PCS-201-010 fibroblasts, p38 MAPK inhibitor SB203580 activated mitophagy to reverse senescence. Induction of replicative senescence was associated with both lysosomal and mitochondrial dysfunction causing increased ROS production and relied on glycolysis for energy source [183]. Inhibition of p38 by SB203580 restored the mitochondrial metabolism by clearing damaged mitochondria by mitophagy thereby reducing dependence on glycolysis. In this study, senescence was monitored solely by SA-β-gal activity and mitophagy was determined by mitochondrial mass and colocalization of LC3 with mitochondria. Moreover, the mechanism by which p38 regulated mitophagy and senescence, and the interdependence of mitophagy and senescence, were not determined.

p38 MAPK activity was elevated in human CD8^+^ T cells re-expressing CD45RA (EMRA) which exhibited several characteristics of senescence but autophagic activity was low [184]. These cells displayed mitochondrial dysfunction and an increase in ROS and relied on anaerobic glycolysis for energy instead of oxidative phosphorylation. The mechanism by which p38 MAPK inhibited autophagy involved interference with ATG9 trafficking by preventing the interaction between the p38 interacting protein with ATG9. Inhibition of p38 MAPK induced mTORC1-independent autophagy by allowing Atg9 trafficking. Restoration of mitochondrial function reversed senescence by providing energy required for cell proliferation via autophagy-mediated degradation and recycling of dysfunctional mitochondria. Since senescent EMRA CD8^+^ cells increase during many diseases, including cancer, aging and autoimmune diseases, p38 inhibitors could be useful to reverse senescence in these diseases. However, the effects of p38 inhibitors on immune function needed to be explored.

A recent study investigated the involvement of p38 MAPK-induced senescence on inflammation. Subtoxic oxidative stress induced senescence in CD4^+^T lymphocytes and was associated with activation of p38 MAPK, altered lysosomal function and impaired mitophagy [185]. In this study, activation of p38 impaired mitochondrial dysfunction by inhibiting mitophagy whereas in the previous study inhibition of mitophagy by p38 caused mitochondrial dysfunction. However, consistent with previous reports [183,184], disruption of mitophagy by p38 activation was responsible for the accumulation of dysfunctional mitochondria, increased ROS production and induction of senescence. Moreover, activation of p38 MAPK in senescent CD4^+^T lymphocytes was also responsible for the release of proinflammatory Th17-type SASP cytokines, such as IL-6, IL-17 and GM-CSF, that are associated with inflammation and bone-resorptive diseases. Thus, p38 inhibitors could be beneficial to attenuate inflammation and restore mitochondrial function.

p38 also regulated autophagy and senescence involving peroxisomes. The peroxin (PEX) receptor PEX5, which regulates peroxisome biogenesis and function, inhibited p38 MAPK activity [186]. PEX5 was downregulated during replicative- and oxidative stress-induced senescence in human primary fetal lung fibroblasts and knockdown of PEX5 induced senescence and impaired autophagic flux. Loss of PEX5 caused activation of p38 which inhibited transcription factor EB (TFEB) by preventing its nuclear translocation. Since TFEB transcribes genes for lysosomal biogenesis, inhibition of TFEB by p38 impaired autophagic flux. PEX5 also induced biosynthesis of taurine and taurine supplementation induced TFEB independent of p38 both in vitro and in vivo. Inhibition of p38 counteracted PEX5 KD-induced senescence by restoring nuclear translocation of TFEB and by cooperating with PEX5-taurine-TFEB signaling. Thus, p38 MAPK inhibitors combined with taurine supplementation could ameliorate age-related lung diseases.

### 5.5. CDK

Since stable cell cycle arrest is the hallmark of senescence, and CDK4/6 inhibitors are FDA-approved for the treatment of hormone receptor-positive, HER2-negative breast cancers, there have been numerous reports on the involvement of CDK inhibitors in senescence [48,86,88,92,187]. In this section, a few articles that associate CDKs with both senescence and autophagy are discussed.

Autophagy and senescence were shown to be part of the same metabolic program known as autophagy–senescence transition or AST [188]. The interaction between the tumor microenvironment and cancer cells is important for tumor progression. When autophagy/mitophagy was induced in *hTERT*-immortalized fibroblasts, they exhibited progressive induction of senescence in the tumor stroma when co-injected with breast cancer MDA-MB-231 cells in mice. The increased catabolism of the autophagic–senescent fibroblasts in the tumor stromal microenvironment generated metabolic fuels, such as lactate and ketone bodies, to promote tumor growth and metastasis in a paracrine fashion independent of angiogenesis. While secretion of SASP cytokines is known to promote tumor growth, autophagy/mitophagy provided metabolic fuels independent of SASP. Induction of autophagy in MDA-MB-231 breast cancer cells, however, suppressed tumor growth due to consumption of cellular compartments.

When senescence was induced in *hTERT*-immortalized fibroblasts or MDA-MB-231 cells by the treatment with a CDK4/6 inhibitor or overexpression of p16^INK4A^, p19^ARF^ or p21, they also exhibited an increase in autophagy, and the phenomenon was termed senescence–autophagy transition or SAT [189]. Induction of senescence in fibroblasts led to the reduction in mitochondrial oxidative phosphorylation, causing a shift towards the glycolytic pathway and promoted tumor growth in a paracrine fashion when co-injected with MDA-MB-231 cells but resulted in tumor suppression in MDA-MB-231 cells. Oxidative stress and starvation induced both autophagy and senescence suggesting that they are metabolically linked.

Osteosarcoma U2OS and prostate cancer PC3 cells were resistant to promyelocytic leukemia (PML)-induced senescence unless CDK4 and CDK6 were depleted or inhibited [190]. Inhibition of CDK4/6 by palbociclib or flavopiridol or knockdown of CDK4/6 also induced autophagy and induction of autophagy enhanced senescence in PC3 cells and tumor xenografts. The mechanism by which CDK4/6 ablation induced senescence involved autophagic degradation of DNA methyltransferase 1 (DNMT1) which is stabilized by CDK4/6-mediated phosphorylation and represses pro-senescence genes via the epigenetic mechanism.

In contrast, autophagy and senescence were inversely related in both mouse and human mammary epithelial cells (MEC) [191]. Overexpression of ErbB2 oncogene is associated with breast cancers and requires cyclin D1 which binds to and activates CDK4/6 for its oncogenic activity. However, when cyclin D1 activity was abrogated in mice, autophagy was upregulated, but ErbB2 failed to induce senescence in vivo. ErbB2 overexpressing MECs could increase cell proliferation, at least temporarily, even when Rb was active. This was due to upregulation of autophagy which maintained cell proliferation in the absence of cyclin D1 since inhibition of both CDK4/6 and autophagy enhanced senescence.

The mechanism by which activation of autophagy attenuated CDK4/6 inhibitors-induced senescence involved ROS and an intact G1/S transition [192]. Inhibition of autophagy potentiated ROS production by CDK4/6 inhibitors and induced senescence both in vitro and in vivo and suppressed tumor growth in breast tumor xenografts. The authors suggested that degradation of ROS by autophagy was responsible for inhibiting senescence induced by CDK4/6 inhibitors. The phosphorylation of Rb by cyclin D-CDK4/6 and cyclin E-CDK2 inactivates Rb allowing cell cycle progression. The cleavage of cyclin E generates a low-molecular-weight cyclin E (LMWE) which hyperphosphorylates RB and is associated with breast cancer. Knockdown of Rb or overexpression of LMWE counteracted palbociclib-induced growth inhibition by reducing ROS production, suggesting the involvement of Rb and cyclin E in CDK4/6 inhibitor-mediated senescence. In fact, not only estrogen receptor-negative breast cancers but also Rb-positive LMWE-negative estrogen receptor-negative breast cancer as well as prostate, ovarian, lung, pancreatic and colorectal cancer cells were sensitive to palbociclib and were further sensitized by autophagy inhibitors. Thus, autophagy inhibitors could enhance therapeutic efficacy of CDK inhibitors in LMWE-negative tumors.

Rb-positive patient-derived osteosarcoma cells were also more sensitive to CDK4/6 inhibitor-induced senescence. Palbociclib primarily induced senescence in osteosarcoma (OS) cells but had a modest effect on autophagy due to compensatory activation of the PI3K/Akt/mTOR pathway which reactivated CDK4/6 [193]. While the PI3K inhibitor voxtalisib alone had little effect on senescence or autophagy, a combination of palbociclib and voxtalisib induced autophagy but had no additional effect on palbociclib-induced senescence. However, the combination was more effective in suppressing tumor growth in both treatment-naïve and treatment-refractory metastatic patient-derived OS models. The dual inhibitors were only slightly effective compared to palbociclib alone in preventing lung colonization of metastatic OS cells. The mechanism of the synergistic cytostatic effect involved palbociclib-induced G1 arrest and senescence and voxtalisib-induced autophagy and was associated with enhanced downregulation of CDK1/2 and Bcl-2 and a decrease in PI3K/Akt/mTOR activity. In this study, induction of autophagy was cytostatic and not cytoprotective. Nevertheless, dual inhibition of CDK4/6 and PI3K/Akt/mTOR signaling could provide a targeted therapy for the treatment of bone cancers.

Cyclin D1 is also frequently overexpressed in gastric cancers but inhibition of CDK4/6 with palbociclib induced senescence in gastric cancer cells regardless of Rb expression but required p53 [194]. However, the ability of palbociclib to induce autophagy was dependent on the status of both Rb and p53. Palbociclib failed to induce autophagy in cells deficient in Rb. While inhibition of autophagosome formation by spatuitin augmented the ability of CDK4/6 inhibitors to inhibit cell proliferation, the number of senescent cells did not increase. The senescent cells exhibited a distinct phenotype of giant multinucleated cells. Knockdown of p53 attenuated palbociclib-induced senescence as well as autophagy. The authors suggested that in gastric cancer cells, the induction of autophagy was not responsible for CDK4/6 inhibitor-induced senescence but rather promoted survival of cells in which CDK4/6 was inhibited.

The same group reported a complex relationship between palbociclib-induced senescence, mTORC1-mediated autophagy and SASP, depending on the status of mTORC1 and autophagy [33]. Induction of senescence by palbociclib in breast cancer MCF-7 and gastric cancer AGS cells was associated with a small decrease in mTORC1 activity as well as autophagy. The mTORC1 inhibitor caused a modest increase in senescence and autophagy and this effect was reversed by the autophagy inhibitor spautin-1. While SASP cytokines released from palbociclib-treated senescent cells inhibited cell proliferation, they increased migration and invasion in non-senescent cells. This effect was increased by the induction of autophagy by the mTORC1 inhibitor and was reversed by the autophagy inhibitor spautin-1. There was, however, significant variability in SASP in MCF-7 versus AGS cells and the autophagy inhibitor spautin-1 did not increase p62, suggesting autophagic flux may be impaired.

## 6. Discussion/Conclusions

Since Narita and colleagues [11] first provided evidence in 2009 that the induction of autophagy by the inhibition of mTORC1/2 also induced senescence, there have been numerous articles that proved or disproved the concept that autophagy and senescence are causally linked and if autophagy promotes or suppresses senescence. A recent article by Attardi and colleagues published in 2026 [160] also reinforced the involvement of mTORC1 in regulating both processes although the mechanisms linking these two processes were different. Several factors decide whether autophagy will promote or prevent senescence, including stages of the disease, types of inducers, duration and intensity of the stress signals, cell type, as well as the timing of autophagy activation and the temporal nature of autophagy. Since kinases play an integral role in regulating both autophagy and senescence, we have analyzed data from both old and recent studies to determine how the status of kinase signaling pathways contributes to this controversy.

Since autophagy maintains organelle and protein homeostasis, which is declined during aging, activation of autophagy is expected to suppress senescence during the normal process of aging or during age-related disorders. A major contributor of both senescence and autophagy is mitochondria-derived ROS. Defects in autophagy/mitophagy cause accumulation of dysfunctional mitochondria generating ROS. Therefore, inhibition of mitophagy/autophagy by mTORC1 is expected to induce senescence [151,152,153,154]. As shown in Figure 2 and Figure 3, activation of autophagy by the inhibition of mTORC1 or activation of AMPK was associated with a decrease in senescence in most cases. However, chronic activation of autophagy is required to support the development of SASP (Figure 2). Moreover, since mitophagy only eliminates new but not old mitochondria [158] sustained mitophagy may, in fact, generate ROS due to accumulation of dysfunctional mitochondria. In that scenario, autophagy may promote senescence.

Cellular context plays a major role in deciding how kinases regulate autophagy and senescence. For example, oncogenes, such as Ras, trigger senescence to serve as a barrier to tumorigenesis. However, when both Ras and the PI3K/Akt/mTOR pathway are active as in the cases with pancreatic cancer, Akt/mTOR antagonized the effects of Ras on autophagy and senescence contributing to the development of pancreatic cancer [146]. In lung adenocarcinoma, oncogenic mutation of KRAS is frequently associated with inactivation of the tumor suppressor p53. Thus, in *KRAS*-mutated lung cancer, inhibition of mTORC1 induced not only autophagy but also induced senescence by preventing MDM2-mediated degradation of p53 (Figure 2), contributing to tumor regression [160]. On the other hand, hyperactivation of PI3K/Akt increased p53 via mTOR-mediated p53 translation and Akt-mediated p53 stabilization in MCF-7 and immortalized fibroblast cells [12,130]. Thus, what determines whether autophagy would promote or suppress senescence across different physiological and pathological conditions needs to be explored.

While the strength of stress signals may decide whether a cell would undergo transient cell cycle arrest, senescence or apoptosis, the intensity and duration of kinase signaling may also dictate cell fate. For example, ERK exhibits biphasic cellular response due to temporal induction of different classes of genes determined by the intensity of ERK activity [121,122]. While high ERK activity was required for senescence induction, low ERK activity was associated with transformation. In addition, the duration of ERK activity determined the maintenance of senescence [106]. The timing of autophagy activation relative to senescence induction is not clearly understood.

Autophagy and senescence are dynamic processes and kinases may regulate different steps of autophagy and senescence differently [110,158]. Kinases may also influence autophagy and senescence by altering the spatial organization of autophagic compartments or altering the cellular localization of their targets. For example, localization of mTORC1 in TASCC enriched in autolysosomes facilitated senescence establishment [145] whereas segregation of damaged mitochondria in special LC3-negative vacuoles by MEK/ERK allowed survival of senescent cells [177]. There is controversy over whether the activation of autophagy is required for the induction, establishment or maintenance of senescence.

Most kinases, such as Akt, mTOR, ERK, JNK and p38, exist as a family and different isoforms may have distinct functions. The crosstalk among various kinases and feedback regulation may also influence how autophagy and senescence are linked. For example, FDA-approved CDK inhibitors used for the treatment of hormone receptor-positive HER2-negative breast cancers are known to suppress tumors by inducing senescence, but their therapeutic efficacy was thwarted due to compensatory activation of the PI3K/Akt/mTORC1 pathway [36,193]. Since these cells had greater dependency on oxidative phosphorylation and glycolysis, metabolic drugs provided a therapeutic opportunity to treat patients resistant to CDK4/6 inhibitors [36].

Methodologies used to detect autophagy and senescence also influenced data interpretation. For example, the activation mechanism and the downstream effectors of mTORC1 and mTORC2 are distinct, and they can influence the activity of each other [54]. Although many of the studies used pharmacologic inhibitors that inhibited both mTORC1 and mTORC2, the conclusions were somewhat biased towards mTORC1 since mTORC1 was identified as master regulator of autophagy [147,151,155,177]. Some of the conclusions regarding the involvement of p38 MAPK relied solely on pharmacological inhibitors, such as SB203580, that may have additional targets [195,196]. While molecular approaches are considered more specific, complete knockout of a gene could activate compensatory signaling pathways whereas overexpression can affect cellular localization and function. Autophagy is often monitored by the increase in LC3-II which may not reflect the complete autophagy process. Kinases may have distinct effects on autophagosome formation versus autophagic flux with different consequences on senescence [151,152,163,181,182,194]. Since SA-β-Gal activity, a widely used marker of senescence, monitors lysosomal function, it may also be affected during autophagy [6]. Kinases may also regulate early and late stages of senescence differently. For example, inhibition of mTOR [131,132,133] or JNK [109] could inhibit SASP but not cell cycle arrest. Thus, if cell cycle arrest is used to monitor senescence, inhibition of a kinase may not show any effect. In addition, the composition of SASP may also be altered, and cellular functions will depend on which SASP factors are secreted [134,174,185]. In addition, SASP secreted factors may exert pro-tumorigenic effects in stromal cells in a paracrine manner but inhibit endogenous cell proliferation [166]. Thus, proper methodologies and careful evaluation of data are required to derive at appropriate conclusions.

There have been numerous attempts to target autophagy and senescence to improve therapy of many diseases, including but not restricted to aging and cancer. Since kinases play a central role in orchestrating both autophagy and senescence, they could provide important targets for therapy. As discussed, many factors including cellular context, inducers of autophagy or senescence as well as methodologies used to monitor autophagy and senescence could affect conclusions drawn from various studies and much more work needs to be done to resolve these issues. A recently published excellent review article discussed how the mTORC1/AMPK signaling pathway could be targeted for therapeutic benefits [25].

A major challenge in targeting kinases, however, is crosstalk and feedback regulation among various signaling pathways. Future studies should apply a systems biology approach to generate a network connecting kinase signaling to the downstream execution of both autophagy and senescence (e.g., SASP regulation and metabolic rewiring) to identify points of clinical interventions. In addition, most of the earlier studies focused on macroautophagy which causes non-specific bulk-degradation of organelles. How kinases drive senescence via selective autophagy, such as mitophagy, lipophagy and chaperone-mediated autophagy, should also be explored. Studies conducted by Lee et al. demonstrate how selective autophagy of specific proteins regulates different steps of senescence to acquire the final stage of senescence. Similar network analyses should include kinase signaling and should be extended to different senescence inducers and disease models [28]. Once experimentally validated, biomarkers could be developed for patient stratification. Thus, a thorough understanding of the mechanisms by which kinases regulate the complex interplay between autophagy and senescence is essential prior to exploiting them as therapeutic targets.

## Figures and Tables

**Table 1:** Regulation of senescence by mitogen-activated protein kinase.

Senescence Inducers	Cell Line/ Mice	Expression Treatment	Senescence Marker	Major Findings	Ref.
H-RasV12	IMR90, *p53*^−/−^ & *INK4A*^−/−^ MEF	WT & mutant RAS, CA-MEK1, PD098059	Altered morphology, ↑p16, p53, p21 & SA-β-Gal	Senescence is dependent on the upregulation of p16 & p53/p21 via MEK independent of PI3K/Akt.	[101]
CA-Raf1	IMR90	CA-p16^INK4A^ E6, PD98059	Altered morphology, ↑p16 & SA-β-Gal	Senescence is dependent on p16 upregulation via Raf/MEK/ERK pathway independent of p53/p21.	[102]
CA-MEK	HIEC, IEC-6	WT & CA-MEK1	Altered morphology, ↑p16, p53, p21 & SA-β-Gal	CA-MEK induces senescence in non-transformed IEC-6 cells via upregulation of p53/p21 & p16 but promotes growth in immortalized IEC-6 cells via upregulation of ERK1b.	[103]
H-RasV12	IMR90, HMEC, *ERK2*^−/−^ MEF	hTERT, E1A & E6/E7 OE, ERK1 & ERK2 KD U0126, AZD6244	Altered morphology, ↑p16, p53, p21 & SA-β-Gal	Senescence is induced via activation of ERK1/2 and proteasome-mediated selective protein degradation.	[104]
H-RasV12	MEF	ERK1 & ERK2 KD	↑p53, p21, p16, γH2AX, & SA-β-gal	ERK2 but not ERK1 induces senescence via transcriptional and translational upregulation of p19^ARF^ and p16^INK4A^.	[105]
H-RasV12	IMR90, MRC5, KP4 pancreatic cancer cells	SCH772984, AZD6244	↑p53, p21, SA-β-gal, DNA damage foci, & SASP	Duration and intensity of ERK signaling decides commitment to senescence by mediating chromatin opening, after which ERK activity is no longer needed.	[106]
JNK deficiency	*JNK1*^−/−^*JNK2*^−/−^ mice		Altered morphology, ↑p53, p21 & SA-β-gal	JNK deficiency induces p53-dependent senescence and is associated with decrease in AP1 transcription factors.	[107]
JNK inhibitor Irradiation	MCF-7 breast cancer, H460 lung cancer, & HEF	JNK1, Bcl-2 & p53 KD, WT & mutant Bcl-2, SP610025, PD98059, SB203580, Pifithrin-α	Altered morphology, ↑p53, p21 & SA-β-gal	JNK KD/inhibition induces senescence via dephosphorylation of Bcl-2, increase in ROS, activation of DDR and increase in p53.	[108]
Irradiation, H_2_O_2_, etoposide	IMR90	JNK1, JNK2 & T53BP1 KD, SP610025, Trichostatin A	↑γH2AX, p21, ↓phospho-Rb	Activation of JNK by ROS causes accumulation of cytoplasmic chromatin fragments and triggers SASP through cGAS-STING pathway.	[109]
Doxorubicin, Mitoxantrone	U2OS, H1299, OVCAR-8 and HUVEC cells	Jun KD, AP1 construct, SP610025, PD98059, SB203580	↑p53, p21, γH2AX, SA-β-gal & SASP	JNK and ERK temporally regulate senescence induced by topoisomerase II inhibitors. Early JNK activation initiates senescence via AP1/cJun and late JNK/ERK activity controls SASP.	[110]
Short telomere, H_2_O_2_, CA-Raf	WI-38 & MRC5 fibroblasts	DN-MKK6, CA-Raf Anisomycin, SB203580, PD98059	↑p16, p21 and SA-β-gal	Chronic p38 activation is responsible for oncogene-induced, oxidative stress-induced & telomere-dependent and independent senescence.	[111]
Ionizing radiation	MRC5, MEF, *CDKN1A* KO MEF	p38, p53 & p21 KD, SB203580	↑p53, p21, γH2AX, SA-β-gal & SAHF	Activation of p38 by p21 during DNA damage causes mitochondrial dysfunction and ROS production. The positive feedback loop between p21 and ROS is sufficient to maintain senescence phenotype.	[112]
X-irradiation, CA-p38, H-RasV12	HCA2, WI-38, MBA-MB-231	p38⍺ & RelA KD, CA-MKK6, GSE22, SB203580	↑SA-β-gal & SASP	High p38 activity is sufficient to induce SASP and is repressed by p53. DNA damage, Ras and CA-p38 require NF-κB for SASP induction.	[113]
Doxorubicin, TPA	Keratinocytes, HCA2 & A-431 cells, p16-MR mice	TPA, SB203580	↑p16, SA-β-gal & SASP	Activation of p38 & ERK contributes to promotion but not initiation of skin cancer via induction of SASP.	[114]
Doxorubicin, H_2_O_2_, Serial passaging	IMR90	ACC1 & FAS KD, TOFA, SB203580	↑p16, γH2AX & SA-β-gal, ↓Phospho-Rb	Activation of p38 contributes partly to ACC1 KD-induced senescence by suppressing SA-β-gal but does not restore cell proliferation.	[115]
hTERT KD	Lung cancer A549 & human umbilical cord fibroblast HUC-F2 cells	TAK1, TAB1 & DN-MKK6, DN-p38⍺, hTERT KD, SB203580, U0126, Anisomycin	↑SA-β-gal	p38 activation induces replicative senescence by repressing hTERT transcription.	[116]
Lamin B1 OE, H_2_O_2_	A-T fibroblasts, A-T lymphoblasts	Lamin B1 OE & KD, p38 KD, KU-55933, SB203580, Anisomycin	↑SA-β-gal & SAHF	Activation of p38 by oxidative stress induces senescence via phosphorylation & stabilization of lamin B1 independent of DDR.	[117]
ATM knockout	ATM^−/−^, p53^−/−^, ATM^−/−^ p53^+/−^, Lung fibroblasts	KU60019, RU.521, H151, PD169316	↑p53, p21, p16, γH2AX, SA-β-gal, & SASP	p38 activation induces senescence in ATM KO murine fibroblasts by inducing p53/p21, p16 and by the amplification of interferon signaling via collaboration with cGAS-STING pathway.	[118]
H-RasV12	BJ, WI-38, IMR90, p38δ^−/−^ MEFs	CA-Raf1, WT & mut MKK; MEK, AP1, ETS & p38δ, & cJun KD, U0126	↑SA-β-gal, No effect on p53, p21 & p16	Increased expression and activation of p38δ by Raf-1–MEK–ERK–AP-1/Ets pathway mediates Ras-induced senescence independent of p53, p21 & p16.	[119]

**Abbreviations**: ACC1: Acetyl-CoA carboxylase 1; ATM: Ataxia-telangiectasia mutated; CA: Catalytically active; cGAS/STING: Cyclic GMP-AMP synthase (cGAS)/Stimulator of interferon genes (STING); DDR: DNA damage response; HCA: Human colon carcinoma; HEF: Human embryonic fibroblasts; HMEC: Human mammary epithelial cells; hTERT: Human telomerase reverse transcriptase; IEC: Intestinal epithelial cells; HIEC: Human IEC; KD: Knockdown; MEF: Mouse embryo fibroblasts; OE: Overexpression; ROS: Reactive oxygen species; SAHF: Senescence-associated heterochromatin foci; SASP: Senescence-associated secretory phenotype; TPA: 12-O-tetradecanoylphorbol-13-acetate.

**Table 2:** Regulation of senescence by mTORC1/AMPK pathway.

Senescence Inducer	Cell Line/Mice	Expression Treatment	Senescence Marker	Major Findings	Ref.
PTEN loss	MEF; Pten^pc−/−^ Trp53^pc−/−^, Pten^pc−/−^Trp53^pc−/−^ mice	PTEN KD	↑p53, p21, SA-β-gal	Acute loss of PTEN induced senescence by stabilizing p53 via p19^ARF^.	[129]
PTEN loss	PTEN^−/−^ MEF, PC3, LNCaP, DU145 cells, Pten^−/−^ & Pten^−/−^p19^ARF−/−^ mice, PC xenograft	PTEN KO, PTEN inhibitor VO-OHpic, Rapamycin, RAD001	↑p53, p21 & SA-β-Gal	PICS is distinct from OIS and did not involve DDR but required p53 stabilization and mTORC1-mediated p53 translation.	[13]
PTEN loss	MCF-7, PTEN^−/−^ MEF, PTEN^−/−^ mice	WT & mut p53, Myr-Akt & mTOR; PTEN, S6K1/2, 4EBP1, Akt & mTOR KD	↑p53, p21, SA-β-Gal & *γ*-H2AX	PICS was dependent on mTORC1/2- mediated phosphorylation of p53 and was independent of DDR, ROS, S6K1/2 & 4EBP1.	[130]
Hyperactive Akt H-RasV12	hTERT immortalized BJ human fibroblasts	PIK3CA^E545K^ & Myr-Akt; PTEN & p53 KD Rapamycin	↑p53, p21, SA-β-Gal & SASP	AIS was dependent on mTORC1-mediated p53 translation and Akt-mediated p53 stabilization. It did not involve DDR and induced SASP which was distinct from OIS-induced SASP.	[12]
Irradiation, Ras, Na butyrate, MKK6EE, doxorubicin	Human fibroblasts, Mammary epithelial cells, prostate cancer cells & PC3 xenograft	mTOR, raptor, S6K & IL1A KD MEE6EE & 4EBP1 Rapamycin	↑SASP	mTOR increased SASP by enhancing IL1A translation & increase in NF-κB transcriptional activity. Rapamycin inhibited SASP cytokines but did not reverse IR-induced cell cycle arrest.	[131]
Irradiation, Ras, Replication exhaustion	IMR90, BJ & HFFF2 fibroblasts, T47D breast cancer & 5PT squamous carcinoma cells	mTOR, ZFP36L1 KD DN-4EBP1, MAPKAP2 & & CA-ZFP36L1 Rapamycin, NVP-BEZ235, Torin 1, MK2 inhibitor III	↑p16, p21, SA- β-gal & SASP	mTORC1 induced SASP by increasing translation of MAPKAPK2 (MK2) which phosphorylated & inactivated ZFP36L1 causing stabilization of SASP factors.	[132]
Irradiation, Ras, Replication exhaustion	MRC5 & PGC-1β-/- MEF Young & old C57/BL6 mice	CA-Rheb (N153T) PGC-1β & mTOR KD KU55933, rapamycin	↑p16, p21, SA-β-gal & SASP	mTORC1 promoted SASP via PGC-1β-dependent mitochondrial biogenesis which coordinated with ROS-induced DDR.	[133]
Doxorubicin	HUVEC & HCC cells, C57Bl/6J & IL6 KO mice	E4ORF1, Myr-Akt & DN-IKK⍺ Rapamycin, SB203580	↑p16, p21, SA-β-gal & SASP	Doxorubicin induced atypical SASP via p38 activation. mTORC1 activation switched acute SASP to sustained SASP independent of NF-κB.	[134]
Cortactin depletion	Patient-derived melanoma, CTC lines, A375, BJ fibroblasts Mel-167 & A375 xenografts	CTTN OE; CTTN, mTOR, & p53 KD Rapamycin, Torin 1	↑p53, p21, SA-β-gal & SASP	Cortactin deficiency induced senescence via mTORC1-mediated p53 phosphorylation which in turn induced p21 and caused ROS-mediated activation of p53 forming a feedback loop to maintain a stable senescent phenotype.	[135]
AMPK activation, Replication exhaustion	IDH4, WI-38 & IMR90	CA-AMPK & DN-AMPK, AICAR, Antimycin A, Azide	↑p16 & SA-β-gal	Activation of AMPK induced senescence through inhibition of cytoplasmic export of HuR decreasing the stability of cyclin A & cyclin B1.	[136]
Glucose limitation, Replication exhaustion	p53 WT, p53^−/−^, p53^+/−^ & p53^S18A/S18A^ MEF	CA-AMPK, DN-AMPK AICAR, rapamycin	↑p53, p21 and SA-β-gal	AMPK induced p53-dependent senescence by phosphorylating p53 to maintain viability of cell cycle arrested cells.	[46]
Oncogenic Ha-RasV12	IMR90	p53, E7, p16^INK4A^ & RISP KD Rotenone	↑p16, p21, *γ*-H2AX & SA-β-gal	Mitochondrial dysfunction contributed to OIS via activation of AMPK and increased ROS production.	[137]
Dysfunctional mitochondria, lack of pyruvate, Irradiation	IMR90, BJ1 & mitochondria-depleted rho0 cells, POLGD257A mice	SIRT3, AMPK & p53, & HSPA9 KD, DN-AMPK 2-DG, pyruvate, rotenone, antimycin A	↑p16, p53, p21, SA-β-gal, ↓LMNB1 Atypical SASP	MiDAS was mediated via AMPK/p53 and was associated with distinct SASP due to inhibition of NF*κ*B by p53.	[138]
MNU	661w RDY rats	Compound C, Hyperoside	↑p16, p21, *γ*-H2AX, SA-β-gal & IL6	MNU-induced senescence via AMPK/ULK1 pathway.	[139]

**Abbreviations**: AIS: Akt-induced senescence; CTC: Circulating tumor cells; CTTN: Cortactin; HCC: Hepatocellular carcinoma; IL1A: Interleukin-1α; MiDAS: Mitochondrial dysfunction-associated senescence; OIS: Oncogene-induced senescence; Phosphatidylinositol-4,5-bisphosphate 3-kinase catalytic subunit-α; PICS: PTEN-loss-induced cellular; PIK3CA: PI3K catalytic subunit; PTEN: Phosphatase and tensin homolog deleted on chromosome ten; RDY; Retinal dystrophy; RISP: Rieske iron sulfur protein; WT: Wild-type.

## Data Availability

No new data were created or analyzed in this study. Data sharing is not applicable to this article.

## Abbreviations

The following abbreviations are used in this manuscript:

3-MA	3-Methyl adenine
4E-BP1	Eukaryotic translation initiation factor 4E binding protein 1
ACC1	Acetyl-CoA carboxylase 1
AIPS	Autophagy impairment-induced senescence
AIS	Akt-induced senescence
AMBRA1	Activating molecule in beclin 1-regulated autophagy protein 1
AMPK	AMP-activated protein kinase
ARF	Alternative reading frame
ASAP	Acute stress-associated phenotype
AST	Autophagy-senescence transition
Atg	Autophagy-related proteins
ATM	Ataxia-telangiectasia mutated
CA	Constitutively active
CDK	cycle-dependent kinases
cGAS/STING	Cyclic GMP-AMP synthase (cGAS)/Stimulator of interferon genes (STING)
CMA	Chaperone-mediated autophagy
CSF	Colony stimulating factor
CTC	Circulating tumor cells
DDR	DNA damage response
DN	Dominant negative
DNMT1	DNA methyltransferase 1
DRP1	Dynamic-related protein 1
EC	Endothelial cells
eIF3	Eukaryotic initiation factor 3
ERK	extracellular signal-regulated kinase
HDAC1	Histone deacetylase 1
IEC	Intestinal epithelial cells
IL	Interleukin
JNK	c-Jun amino (N)-terminal kinase
KD	Knockdown
KEAP 1	Kelch-like ECH-associated protein 1
LMWE	Low-molecular-weight cyclin E
MAPK	Mitogen-activated protein kinases
MDM2	Murine Double Minute 2
MEC	Mammary epithelial cells
MEF	Mouse embryo fibroblasts
MEK	Mitogen-activated protein kinase kinase
MiDAS	Mitochondrial dysfunction-associated senescence
MK2	MAPK-activated protein kinase 2 or MAPKAPK2
mTOR	mechanistic target of rapamycin
NDP52	52 kDa nuclear dot protein
NF-κB	Nuclear factor κB
Nrf2	Nuclear factor erythroid 2-related factor 2
NF1	Neurofibromin 1
OIS	Oncogene-induced senescence
OPTN	Optineurin
OS	Osteosarcoma
PCC	Pancreatic cancer cells
PDK1	3-phosphoinositide-dependent kinase 1
PEX	Peroxin
PI3K	Phosphatidylinositol 3-kinases
PICS	PTEN-loss-induced cellular
PIP3	Phosphatidylinositol 3,4,5-trisphosphate
PKC	Protein kinase C
PKD	Protein kinase D
PSC	Pancreatic stellate cells
PTEN	Phosphatase and tensin homolog deleted on chromosome ten
PTTG1	Pituitary tumor-transforming 1
RIS	Ras-induced senescence
ROS	reactive oxygen species
RPE	Retinal pigment epithelial
RS	Replicative senescence
S6K	ribosomal S6 kinase
SA	Senescence-associated
SAHF	Senescence-associated heterochromatin foci
SAPD	Senescence-associated protein degradation
SASP	Senescence-associated secretory phenotype
SAT	Senescence-autophagy transition
SeRP	Senescence restriction point
SIPS	Stress-induced premature senescence
TASCC	TOR-autophagy spatial coupling compartment
TFEB	Transcription factor EB
TIS	Therapy-induced senescence
TMZ	Temozolomide
TNIP1	TNFAIP3 interacting protein 1
TSC2	tuberous sclerosis complex 2
ULK	Unc51-like kinase
UVRAG	UV radiation resistance-associated gene
